# Mapping human dispersals into the Horn of Africa from Arabian Ice Age refugia using mitogenomes

**DOI:** 10.1038/srep25472

**Published:** 2016-05-05

**Authors:** Francesca Gandini, Alessandro Achilli, Maria Pala, Martin Bodner, Stefania Brandini, Gabriela Huber, Balazs Egyed, Luca Ferretti, Alberto Gómez-Carballa, Antonio Salas, Rosaria Scozzari, Fulvio Cruciani, Alfredo Coppa, Walther Parson, Ornella Semino, Pedro Soares, Antonio Torroni, Martin B. Richards, Anna Olivieri

**Affiliations:** 1 Dipartimento di Biologia e Biotecnologie “L. Spallanzani”, Università di Pavia, Pavia, Italy; 2 School of Applied Sciences, University of Huddersfield, Queensgate, Huddersfield, UK; 3 Dipartimento di Chimica, Biologia e Biotecnologie, Università di Perugia, Perugia, Italy; 4 Institute of Legal Medicine, Medical University of Innsbruck, Innsbruck, Austria; 5 Department of Genetics, Eötvös Loránd University, Budapest, Hungary; 6 Unidade de Xenética, Departamento de Anatomía Patolóxica e Ciencias Forenses, and Instituto de Ciencias Forenses, Facultade de Medicina, Universidad de Santiago de Compostela, Santiago de Compostela 15782, Galicia, Spain; 7 Dipartimento di Biologia e Biotecnologie “Charles Darwin”, Sapienza Università di Roma, Rome, Italy; 8 Dipartimento di Biologia Ambientale, Sapienza Università di Roma, Rome, Italy; 9 Forensic Science Program, The Pennsylvania State University, University Park, Pennsylvania, USA; 10 CBMA (Centre of Molecular and Environmental Biology), Department of Biology, University of Minho, Campus de Gualtar, 4710-057 Braga, Portugal

## Abstract

Rare mitochondrial lineages with relict distributions can sometimes be disproportionately informative about deep events in human prehistory. We have studied one such lineage, haplogroup R0a, which uniquely is most frequent in Arabia and the Horn of Africa, but is distributed much more widely, from Europe to India. We conclude that: (1) the lineage ancestral to R0a is more ancient than previously thought, with a relict distribution across the Mediterranean/Southwest Asia; (2) R0a has a much deeper presence in Arabia than previously thought, highlighting the role of at least one Pleistocene glacial refugium, perhaps on the Red Sea plains; (3) the main episode of dispersal into Eastern Africa, at least concerning maternal lineages, was at the end of the Late Glacial, due to major expansions from one or more refugia in Arabia; (4) there was likely a minor Late Glacial/early postglacial dispersal from Arabia through the Levant and into Europe, possibly alongside other lineages from a Levantine refugium; and (5) the presence of R0a in Southwest Arabia in the Holocene at the nexus of a trading network that developed after ~3 ka between Africa and the Indian Ocean led to some gene flow even further afield, into Iran, Pakistan and India.

Low-frequency mitochondrial (and Y-chromosome) lineages with a relict distribution can be disproportionately informative about deep events in human prehistory. Mitochondrial DNA (mtDNA) haplogroups N1a1a and X, which have both been recovered from prehistoric remains as well as from living people, are good examples^1^,^2^,^3^. Another such lineage, with a very different distribution, is mtDNA haplogroup R0a, although to date it has never been recovered from prehistoric remains so we are entirely reliant on the modern diversity to draw conclusions about its history. R0a is unique in reaching very high frequencies in the Arabian Peninsula, and is also common on the far side of the Bab el-Mandeb strait (or “Gate of Tears”), in the Horn of Africa, along with several other haplogroups of Eurasian origin.

More generally, the Horn of Africa is exceptional in harbouring very high mtDNA haplogroup diversity^4^, and populations in the Horn have significant non-autochthonous African ancestry across the genome^5^,^6^,^7^,^8^,^9^,^10^. Recent studies of complete human genomes have concluded that this 30–50% of non-African legacy in Cushitic- and Semitic-speaking populations is the result of admixture from Arabia beginning ~3,000 years ago (3 ka)^11^,^12^, at a time when common cultural features developed across the Horn and southern Arabia^13^, suggesting a link with the origin of the Ethiosemitic languages^14^. However, others have argued that such autosomal dating needs to be treated with considerable caution^10^,^15^. Moreover, some have also proposed that the source for the Horn lineages was in the Levant rather than Arabia^10^,^11^, whilst others have provided further evidence in favour of Arabia^15^.

Analyses of the uniparental genetic systems, in particular mtDNA, have suggested much more ancient gene flow into the Horn, from both the Levant and Arabia, although the timing has not been very clearly defined. Haplogroup M1 is thought to have arrived from the Mediterranean some time since the Last Glacial Maximum (LGM)^16^. The North African haplogroup U6a is found at lower levels, and with possibly a different trajectory^16^. Haplogroup N1a1a in the Horn also separated from Arabia in the Late Glacial^3^, and several African subclades of haplogroup R0a and of haplogroup HV1 have been dated to the mid-Holocene^17^,^18^. The Y-chromosome^19^,^20^,^21^ and several genome-wide studies^10^,^15^ have recently supplied further evidence supporting the scenario of ancient migrations from the Arabian Peninsula into the Horn of Africa, well before the spread of agriculture into that region. Fernandes *et al.*^15^ recently estimated the combined Near Eastern/Arabian genome-wide fraction in Ethiopia at almost 40%, closely matching the West Eurasian fraction of 37% in our Ethiopian mtDNA database.

The most prominent Eurasian mitochondrial lineage that is shared across the Horn and Arabia is R0a, which is found at very low frequencies across west Eurasia, but reaches levels of up to 35% in eastern Yemen and up to 15% in some parts of the Horn of Africa facing the Red Sea^9^,^15^,^17^,^18^,^22^,^23^,^24^,^25^,^26^,^27^. It has been thought to have originated in the Near East and to have spread into Arabia at the end of the Pleistocene, albeit with difficulties in defining a source^27^; others have hypothesized a more ancient ancestry within Arabia^28^. This question is of great interest because evidence in favour of deeper Arabian ancestry would imply the existence of refugial areas in Arabia spanning the Last Glacial Maximum, which have been hypothesized but never confirmed^29^. The timing and mode of its subsequent entry into Eastern Africa also remain to be clarified^15^,^27^, as well as its history in Europe^30^,^31^. Here we analyse 205 whole mitogenomes from R0a, and its sister clade R0b, alongside 733 R0a and R0b control-region sequences, in order to address these issues.

## Results

### Deep ancestry of R0a

**Figure 1:**
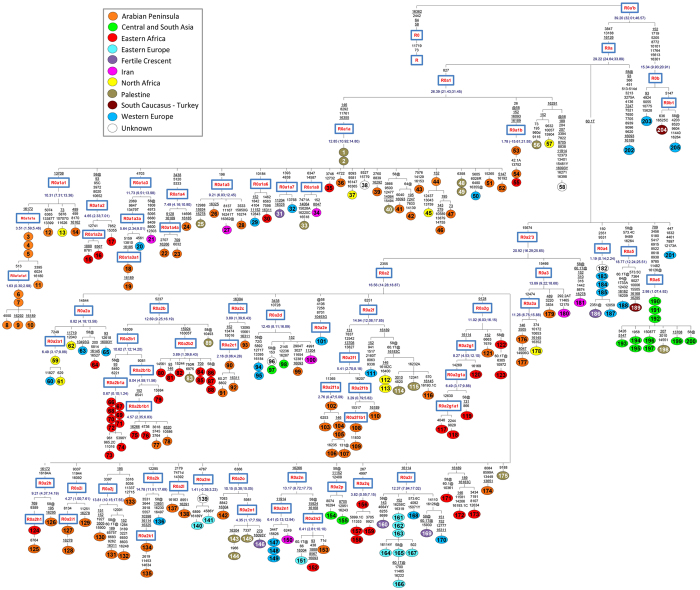
Maximum-parsimony phylogenetic tree of 202 complete mtDNA sequences belonging to haplogroup R0a. Three R0b sequences are also included. Each circle represents a mitogenome and numbers are the same as those in Table S1. Mutations are shown on the branches (relative to rCRS); they are transitions unless the base change is explicitly indicated. Suffixes indicate: transversions (to A, G, C, or T), deletions (d), heteroplasmies (R and Y) and reversions (@). Insertions are also suffixed with a dot followed by a number indicating how many bases were inserted and the inserted nucleotide/s (.1C). Recurrent mutations are underlined. The variation at np 16519, in the number of Cs at nps 309 and 315 as well as the AC indels at nps 515–522 were not included in the phylogeny. All the samples are coloured according to their geographic origin as shown in the legend. ML age estimates are reported in ka for nodes encompassing at least three mitogenomes, except for R0a5 (two mitogenomes), which is extremely rare.

R0a’b (of which R0a forms the major part: Fig. 1; Fig. S1), which dates to ~40 ka using ML, is the sole known sister clade to the major West Eurasian haplogroup HV, with the two together comprising haplogroup R0. R0 branches directly from macro-haplogroup R, which dates to ~59 ka^15^. Although haplogroup R predominates amongst West Eurasians, especially Europeans, continent-specific basal branches are also found amongst South Asians, East Asians, Southeast Asians and Oceanians^32^. Thus whilst haplogroup R is a global non-African founder clade, R0 is primarily West Eurasian.

R0a, dating to ~30 ka using ML (Table 1) falls into at least five major subclades, three (R0a1, R0a2’3 and R0a4) already known^17^,^33^ and two (R0a5 and R0a6) newly defined here (Fig. 1). Two further basal haplotypes (#201 and #202) are seen in Italy and Spain, respectively. Along with a third lineage basal to R0a1 known from control-region data to occur in Egypt (Fig. S1), and the distribution of the very rare R0b, these might suggest a pre-LGM Mediterranean/Near Eastern source for R0a and R0a1, 25–40 ka. Alternatively, they might represent relicts of Late Glacial or postglacial dispersals around the Mediterranean.

R0a4, R0a5 and R0a6 are all rare. A survey of the R0a5 HVS-I motif indicates a wide distribution across the Near East and Europe (Table S2), and a deep Glacial ancestry (36.9 ± 14.1 ka with HVS-I; the two mitogenomes diverge at 18.8 ± 6.6 ka). A similar assessment for haplogroup R0a6 is more difficult, because its only control-region mutation is the reversion of the 16126 transition, but its distribution appears to be mainly restricted to Pakistan (mainly but not exclusively Kalash), with Palestinian, Iranian and Italian singletons (see also Fig. S1). Given its prevalence in the Kalash, we may hope that future elucidation of this lineage may help shed light on the origins of the Kalash people.

In contrast, the frequency and distribution for R0a4 cannot be assessed from published datasets because it lacks any diagnostic control-region mutations. With this limitation in mind, Fig. 1 and Table S1 indicate that R0a4 encompasses mainly mitogenomes from Western Europe, Spain in particular, but also Iraq.

### An Arabian source for the major R0a lineages

**Figure 2:**
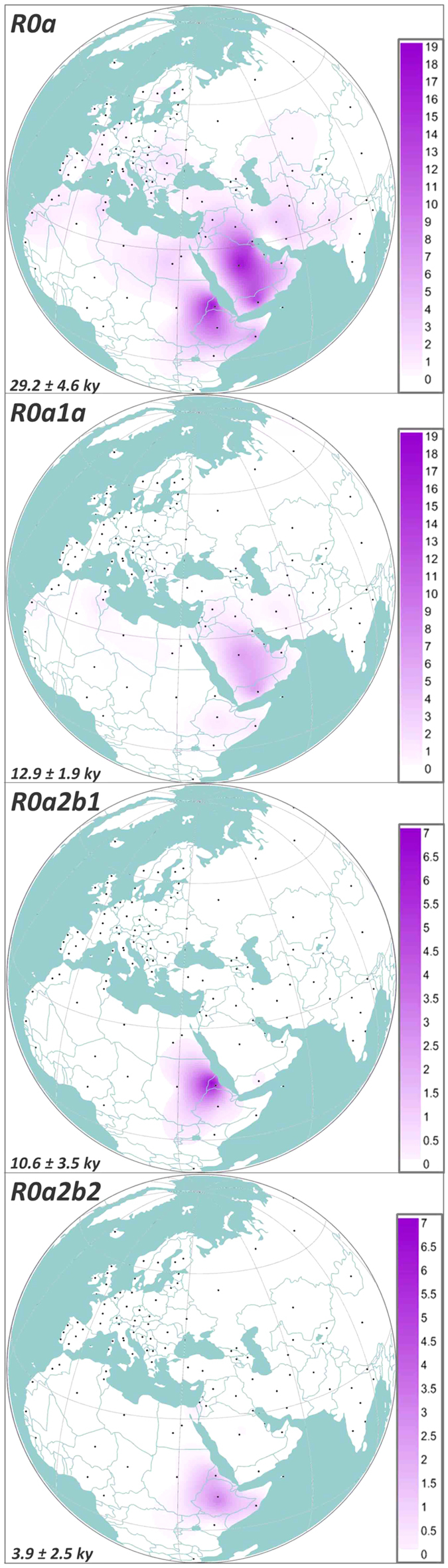
Spatial frequency distribution maps of haplogroups R0a, R0a1a, R0a2b1 and R0a2b2. Dots indicate the geographical locations of the surveyed populations. Population frequencies (%) correspond to those listed in Table S2. The extremely high frequencies of R0a and R0a1a in the Socotra sample (38.5% and 24.6%, respectively) were not included in order to provide a correct representation of the much lower frequencies in the regions surrounding the island. We constructed spatial frequency distribution plots with the program Surfer 9 (Golden Software, http://www.goldensoftware.com/products/surfer).

The great majority of R0a mitogenomes cluster within R0a1 and R0a2’3, dating to the LGM (~26 ka and 21 ka, respectively), each mainly represented by a single star-like subclade, R0a1a and R0a2. These subclades both coalesce to the Late Glacial: ~13 and 17 ka (Table 1). These are the two major expansion lineages in R0a, but although widespread, they are both overwhelmingly seen in Arabia, especially Yemen (Fig. 2). However, R0a1 also includes R0a1b, comprising mainly lineages from Arabia, and several possibly related lineages including a Bedouin from Arabia and a Moroccan. Given that the former have an Arabian origin and the latter are also from Arab-speaking populations, that probably spread from Arabia during the Muslim conquests, the whole of R0a1 seems likely to have an Arabian origin, dating back to at least 26 ka and thus spanning the LGM. This implies that the several Iranian lineages and a single Syrian lineage within R0a1a were derived from an Arabian source. This is supported by the HVS-I network, in which Iranian lineages broadly represent a small subset of Arabian R0a1a diversity (Fig. S1). This is also the case for the few Syrian and Iraqi lineages, and the single branch shared by two Druze individuals is very recently diverged. Moreover, an overall ρ estimate for Fertile Crescent lineages in the HVS-I network for R0a1a, as a simple, unbiased measure of diversity, is only 64.4% of that for Arabian lineages. Thus R0a1 most likely entered Arabia by 26 ka, with the few northern Near Eastern lineages due to recent gene flow from Arabia into the Fertile Crescent. We need to recall this when we consider the founder analysis, below.

Similarly, R0a2’3, at ~21 ka, most likely has an Arabian ancestry. R0a3 is a minor Late Glacial Arabian subclade that sits alongside a paraphyletic Iranian lineage (shared with an Egyptian in the HVS-I dataset). As with R0a1a, Iranian HVS-I lineages within the major R0a2 are broadly a subset of Arabian diversity, with a number of ancestral haplotypes at elevated frequencies (Fig. S1). This may be explained by sporadic gene flow across the Gulf, but some Iranian lineages (along with lineages found further east in Pakistan) may also represent gene flow along the maritime trading networks which intensified in the mid- to late Holocene^34^. There is also a subclade, R0a1a1a, dating to ~3.5 ka (part of a larger clade, R0a1a1, that is also largely restricted to Yemen, dating to 10.3 ka), associated with the settlement of the island of Socotra, which may also have been part of a wider process of increased maritime activity and exchange^35^.

Similarly to R0a1a, if we examine R0a2 lineages from the Levant as a potential source pool, although some are ambiguous, more than a third of the R0a Druze in the HVS-I network (Fig. S1) belong to a derived largely European subclade (R0a2r), dating to ~12 ka (younger than the Arabian expansions); one belongs to a European cluster; and several to Arabian clusters. Again, of four Syrian lineages in the database, one belongs to the European/Druze R0a2r, one to the diverse Arabian subclade R0a2f (which also includes more than a third of Iraqi lineages at its tip), and one to R0a1a7, the most frequent in Yemen according to the HVS-I network, with derived lineages in Pakistan and possibly also Oman (Fig. S1). This phylogeographic pattern is markedly distinct from that in R0a5, for example. A comparison of overall ρ in HVS-I for putative R0a2 lineages (although much less clearly distinguished in the network) shows that the ρ value for the Fertile Crescent is below (albeit closer: 95.6%) that of the Arabian lineages. Again, the best explanation is an Arabian source for the Levantine lineages, in some cases as a result of sporadic gene flow, but for the majority due to Late Glacial expansions through the Levant into Mediterranean Europe, as we discuss further below. This once again suggests a Glacial arrival in Arabia, by 26 ka, although in this case the existence of the Levantine/European R0a2r subclade may suggest that we should not completely rule out a source in a Levantine refugium and Later Glacial expansions into Arabia as an alternative possibility.

With this caveat, this overall pattern strongly suggests that R0a1 and R0a2’3 both entered Arabia before or around the LGM and that the R0a1b/R0a1* and R0a3/R0a2’3* lineages are relicts that were not caught up to the same extent in the Late Glacial expansions that followed the LGM. This conclusion is further supported by the Bayesian skyline plots (BSPs) and reciprocal founder analyses detailed below.

### Expansions of R0a1 and R0a2’3 lineages

The conclusion is strengthened by the distribution of the remaining lineages within each subclade. R0a1a encompasses at least eight major subclades (R0a1a1–8; R0a1a5–8 newly reported), and many paraphyletic lineages. Levantine lineages belong mainly to Negev desert Bedouin and Palestinians. The Bedouin have an Arabian Peninsula ancestry, and genome-wide PCA and ADMIXTURE analyses indicate that Palestinians too are more similar to Arabian populations than to other Levantine populations, and likely have substantial Arabian ancestry^36^,^37^. There is a single small Ethiopian subclade, R0a1a2, dating to ~5 ka but diverging directly from the R0a1a root, and several sporadic singleton Horn lineages, but the vast majority of African R0a lineages fall within R0a2.

The larger R0a2 dates to ~16 ka, with 18 derived subclades which coalesce mainly to the Late Glacial, ~13 and 15 ka (Table 1). The Bølling-Allerød interstadial began ~14.7 ka^38^, and is associated with de-glaciation in Europe and a wet phase in the Near East/Arabia, which might have facilitated movements of hunter-gatherers into previously arid areas^39^. There are two major Eastern African subclades, R0a2b and R0a2g, dating to ~13 and ~11 ka respectively, and several minor ones, one of similar age and another of which dates to ~4 ka but again diverges basally from R0a2. There is also a major Late Glacial subclade, R0a2r, found in southern Europeans but with two basal Druze lineages (from Israel and Lebanon); and several very minor subclades pointing to dispersals into Eastern Europe and Iran/Pakistan.

**Figure 3:**
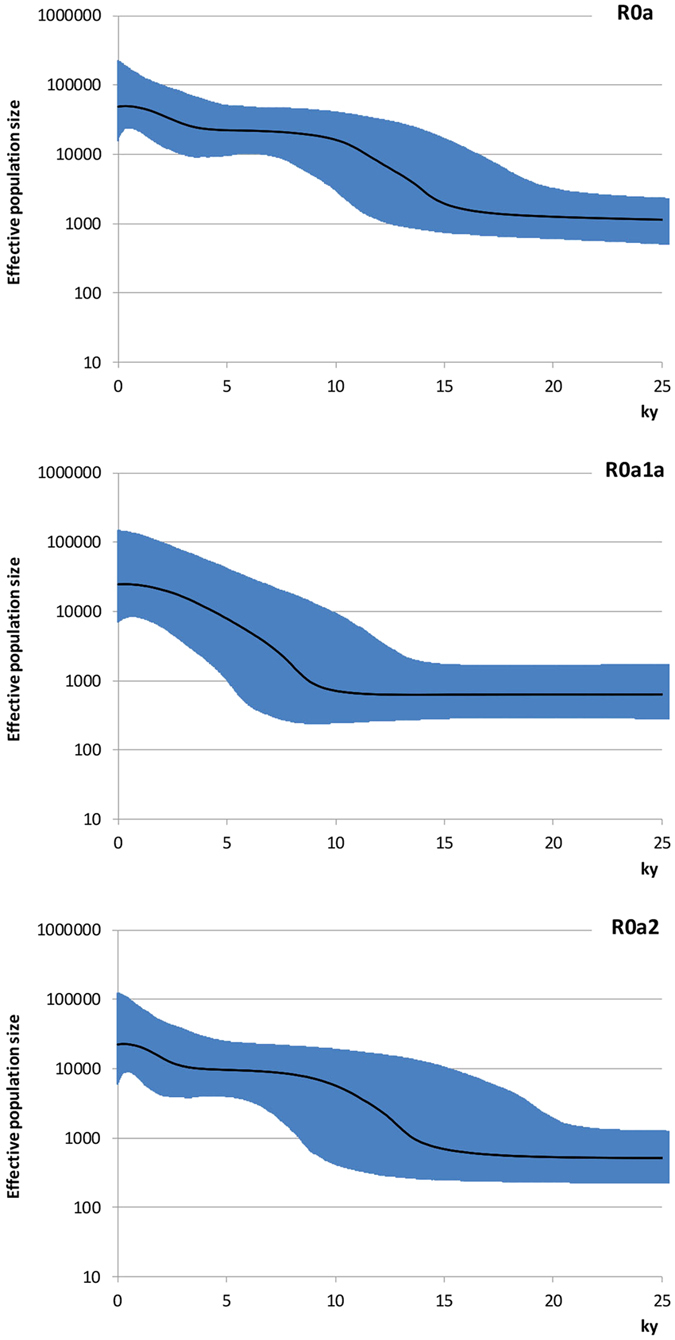
Bayesian skyline plots (BSPs) of haplogroups R0a, R0a1a and R0a2. The thick solid line is the median estimate and the shading shows the 95% highest posterior density limits. The time axis is limited to 25 ka, beyond which the curves remain flat. Hypothetical effective population sizes through time are based on the mitogenomes listed in Table S1.

The BSPs (Fig. 3) show that these coalescences correspond to two major phases of population growth amongst R0a lineages in both the Late Glacial – the Bølling-Allerød interstadial (R0a2) – and the immediate postglacial, after the Younger Dryas (R0a1a). The BSP for R0a as a whole points to a major episode of ~12-fold growth from ~16 ka until ~10 ka, with a more recent episode of ~20-fold growth at ~3 ka. The separate plots show that whilst the growth of R0a2 overlaps with R0a overall, R0a1a was involved in a subsequent population expansion, in the early postglacial warming period following the Younger Dryas glacial relapse, ~11.5 ka. The finding of distinct demographic histories for R0a1a and R0a2 suggests that they may at one time have characterized different populations, possibly even dispersing from separate glacial refugial areas.

BSPs based on geographic origin (Fig. S2) confirmed a primary Bølling-Allerød expansion, with an additional expansion restricted to the Arabian Peninsula ~3 ka (shadowed in Eastern Africa). The plots also suggest that the earliest major signal of Late Glacial expansion was in Arabia, beginning ~17 ka, rather than in the Fertile Crescent (~14 ka), once again supporting an Arabian source. There is no independent expansion signature in Eastern Africa.

### Major dispersal episodes: founder analysis

In order to date and quantify the main dispersal episodes, we performed a founder analysis on the mitogenome data. This identifies “founder sequences” shared between two populations as potential evidence for gene flow between the two populations. In this case, however, this poses a problem, since we have seen above that we cannot uniquely identify a source population, and that most if not all of the Levantine and Iranian lineages in the major subclades are likely due to subsequent gene flow. (This is almost certainly the case also for most of the Mediterranean and North African lineages within R0a1 and R0a2’3.) Nevertheless, we performed the analysis assuming a northern source, in order to provide the most conservative estimate for the age of Arabian lineages. Although this assumption almost certainly doesn’t hold for R0a1a and probably also for R0a2, the analysis can still provide a clear picture of the main expansion episodes, to complement the skyline plots.

**Figure 4:**
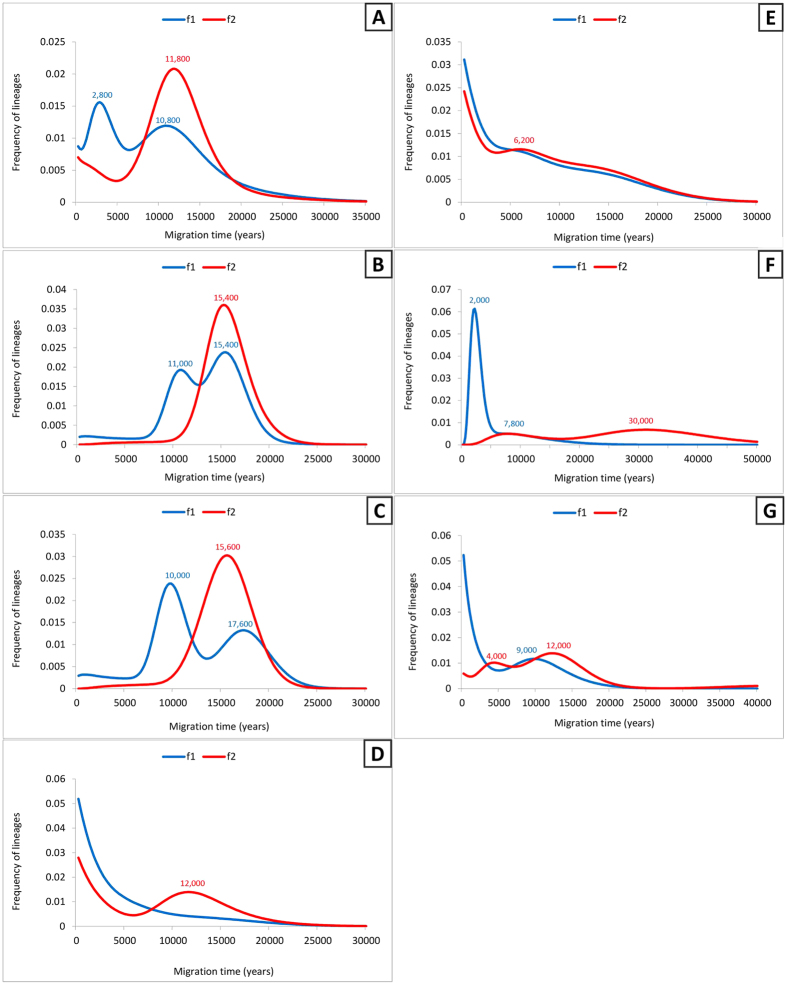
Founder analysis of R0a. Probabilistic distribution of founder clusters across migration times, with time scanned at 200 year intervals from 0 to 50 ka, using *f1* (blue lines) and *f2* (red lines) criteria. (**A**) from the Fertile Crescent, Caucasus, Iran and the Arabian Peninsula to Eastern Africa; (**B**) from the Fertile Crescent and Caucasus to Arabian Peninsula and Eastern Africa; (**C)** from the Fertile Crescent and Caucasus to the Arabian Peninsula; (**D**) from the Arabian Peninsula to the Fertile Crescent, Iran and Caucasus; (**E**) from the Arabian Peninsula to the Fertile Crescent and Caucasus; (**F**) from the Fertile Crescent, Iran, North Africa, the Arabian Peninsula and Caucasus to India and Pakistan and (**G**) from the Fertile Crescent, Caucasus, Iran, North Africa and the Arabian Peninsula to Europe.

We therefore assumed a source in the Fertile Crescent, including the Levant and Iraq, both with and without Iran, in order to explore further the pattern in Arabia and to quantify and date subsequent dispersals into the Horn of Africa, Europe and South Asia, including Arabia in the source when assessing dispersals into Eastern Africa (Tables S3–S9, Fig. 4). We included Levantine Bedouin and Palestinian lineages as part of the Arabian sample, as discussed above.

First, we show Eastern Africa alone as the sink (Fig. 4A and Table S3), with the whole of Southwest Asia as the source. Here there is no Late Glacial peak, but rather a clear signal right at the start of the Holocene with both criteria: 11.8 ka with *f2* and 10.8 with *f1*. With *f2*, this is the sole signal, but with *f1* there is a second, more recent peak at 2.8 ka. The difference occurs in R0a2b, which is classed as a single African founder by the *f2* criterion, whereas R0a2b2 is classed as a distinct founder dating to 2.9 ka with *f1*. This lineage has been elevated to high frequency (10.3–12.5%, the most frequent lineage) in Ethiopian Jews against a genome-wide background that is almost identical to other Ethiopians, and it is not seen in Yemeni Jews, where an Arabian lineage within R0a2c is seen at even higher frequency^22^,^40^ instead. Because of this, despite the superficial confirmation of the ~3 ka migration inferred from autosomal studies, we should be cautious of taking the *f1* result at face value. It may be that this population has subsequently experienced gene flow back towards the Levant, and that this is the reason for identifying the migration with *f1* that is screened out with the more stringent *f2.* However, given the inferences of substantial later northwards gene flow discussed above, we consider *f2* the more plausible criterion for this dataset, at least regarding the settlement of Arabia. Nevertheless, some gene flow ~3 ka is possible, especially given the strong growth signal around this time in the Arabian BSP, and may also be indicated by mtDNA haplogroup HV1 (see Discussion).

We next show the results when Eastern Africa and Arabia are combined into a single sink population (Fig. 4B and Table S4). The *f2* criterion indicates a single Late Glacial expansion at ~15.4 ka, involving all R0a lineages. The *f1* criterion distinguishes an additional more recent, postglacial expansion for R0a1a, ~11.0 ka, but the above discussion has explained why an additional migration is an unlikely scenario in practice. It does highlight, however, that further expansion, involving R0a1a in particular, took place in the postglacial, as also shown in the skyline plots. There is no sign under either criterion of the more recent dispersal at ~3 ka, confirming that, if it occurred at all (and involved R0a), its source was within Arabia and not in the Fertile Crescent.

We next show the results with Arabia alone as the sink, with the Fertile Crescent (excluding Iran) as the source (Fig. 4C and Table S5). Here again we see the major dispersal with the *f2*, ~15.6 ka. This represents our best estimate for the timing of the Late Glacial expansion of R0a. With *f1* we see again both an even earlier Late Glacial peak at 17.6 ka, and an additional episode at ~10.0 ka.

The reciprocal founder analysis, assuming Arabia as source and the Fertile Crescent as sink, including the Levant, Iraq and Iran (Fig. 4D and Table S6), shows a very slight early Holocene peak in *f2* and major peaks towards the present for both *f1* and *f2*. The picture is similar whether or not Palestinians are included within the Arabian source (not shown). Since the peaks are much more recent when Arabia is the source, this implies that any dispersals from Arabia towards the Fertile Crescent must have been much more recent than dispersals in the opposite direction. An analysis that excludes Iran (Fig. 4E and Table S7) differs in detail, yet retains the general features of more recent Holocene peaks especially towards the present for both *f1* and *f2*. These results re-emphasise that the Fertile Crescent R0a variation seen today cannot be the main source for much of the diversity in Arabia, again confirming that Arabia is the most ancient reservoir of R0a variation. This in turn supports the arguments given above that the founder estimates for Arabia are in fact most likely expansion times within the Peninsula rather than dispersals from a Levantine refugium in the north.

Finally, we tested the migrations to South Asia (Fig. 4F and Table S8) and Europe (Fig. 4G and Table S9). As for the Horn of Africa, and unlike for Arabia, we can safely interpret these results straightforwardly in terms of dispersals from an Arabian source. The results of the former shows a small peak ~7.8 ka with both *f1* and *f2* criteria, based on very few sequences, and a stronger signal ~2 ka with *f1*, corresponding to R0a6. The mitogenomes yielding the ~2 ka signal mostly belong to the Kalash community, which is very isolated and carry low diversity of a number of mtDNA lineages of west Eurasian origin^41^. The 2 ka signal transposes to ~30 ka with *f2,* but examination of the tree shows clearly that this is an artefact: the lack of additional lineages deriving from the *f2* founder candidate in South Asia, the low diversity within the Kalash and the presence of a Palestinian lineage in the clade, all point to the more recent introduction of the rare R0a6, suggesting that it may have been insufficiently sampled in Southwest Asia.

The results for Europe also suggest a primary dispersal into Southeast and Mediterranean Europe at the end of the Pleistocene/early Holocene, mainly involving R0a2r, with the signal a little earlier with *f2* than *f1*. This may have been via a Levantine refugium, given the presence of basal Druze lineages in the cluster (and a Syrian in the HVS-I data). It recalls the patterns detected in a much larger fraction of haplogroup J and T lineages that dispersed from an inferred Levantine refugium along the Mediterranean after the LGM^42^. Some lineages may have dispersed later in the Holocene, but this is unclear given the small sample size (R0a occurs amongst Europeans at a rate of only 0.8%).

## Discussion

### Evidence for glacial refugia in Arabia

The earliest settlement of Arabia by modern humans and its role in modern human dispersals out of Africa remain controversial^43^, although the consensus genetic estimate for the timing remains ~50–60 ka. We have argued for a “southern-route” dispersal out of Africa via Arabia at this time, since a Levantine source for all non-Africans would imply that basal non-African mtDNA diversity should be highest in the Near East, whereas the highest diversity is rather seen in South Asia^30^,^44^,^45^. A model of this kind – albeit, inevitably, with further complexity – is supported by the high productivity of ancient coastlines^46^,^47^,^48^. Autosomal dating has been used to suggest an earlier date^49^, and both qualitative arguments^50^ and simulations^51^ have been used to propose that the age of non-African mitogenomes might be older than the ~50–60 ka usually estimated^52^. However, these assertions are based on lines of reasoning that draw their estimates from inappropriately old population splits or ignore the phylogenetic and phylogeographic structure of mtDNA, where inferences are made from a hierarchy of nesting relationships, analogous to a stratigraphy, rather than simple haplogroup ages as often assumed by critics^45^,^53^,^54^. The model of a southern-route dispersal at ~50–60 ka has recently received strong support from an analysis of 104 complete genomes from Arabia^55^. These results are congruent with the most comprehensive mitogenome analyses that also stress the complexity of Arabian demographic history^15^,^56^, and with recent ancient DNA analyses^57^,^58^, although contrary to one rather idiosyncratic reanalysis of mitogenome data that minimises the role of Arabia^59^. Potential earlier dispersals identified from archaeological evidence^51^ therefore seem unlikely to have contributed substantially to the extant gene pool of the region. However, this is a topic that clearly requires much greater discussion, beyond the scope of the present article.

The earliest non-African ancestor of R0a, the root of haplogroup R, dates to ~59 ka, and may (in line with the arguments summarised in the preceding paragraph) have originated in the Gulf Oasis soon after the dispersal of modern humans from Eastern Africa^3^. Its more immediate ancestor, R0a’b, dates to ~40 ka and its earliest branches have a relict distribution around the Mediterranean/Near East. We have identified several new minor sister subclades to the main R0a branches, and these too have a similar distribution.

Nevertheless, multiple lines of evidence suggest that the major R0a subclades had entered Arabia and begun diversifying before the Last Glacial Maximum. This is in accord with evidence from rock art in Northern Arabia that the Neolithic pastoral economy was adopted by hunter–gatherers, rather than introduced by dispersing agriculturalists from the Near East^60^. However, there is little archaeological evidence for the presence of human populations in Arabia across the LGM, when environmental conditions were extremely poor^61^,^62^, suggesting that they survived, if at all, in glacial refugia. Rose^29^ proposed three potential “oases” in Arabia. Most attention has been given to the Gulf Oasis in the east which, as mentioned above, may have incubated early modern humans shortly after their initial move out of Africa. However, there are two further candidates – the South Arabian refugium in the Dhofar highlands and eastern Yemen-Oman coastal zone, and the Red Sea coastal plain^29^. It seems likely that one or both of these were refugia for early Arabian hunter-gatherer groups carrying predominantly R0a1 and R0a2’3, and from which R0a1a and R0a2, in particular, expanded after the LGM. It is tempting to speculate that R0a2’3 may have sheltered in the Red Sea refugium, given the very early postglacial dispersals of R0a2 subclades both into the Horn of Africa and into southern Europe, likely via the Levant. Further work should enable us to test this hypothesis more precisely.

R0a1a began its dramatic expansions ~12 ka and is now seen mainly in the southern part of the Arabian Peninsula, such as Yemen and the island of Socotra, where it displays a more recent frequency peak approaching 40%^35^. However, the first major expansions in Arabia were earlier, in the early Late Glacial period, and involved R0a2. Intriguingly, both expansions predate the early Holocene onset of pluvial conditions in the Peninsula^63^, and perhaps involved coastal regions now under water. Furthermore, R0a2 lineages expanded much further afield, across the Red Sea and into the Horn of Africa, in the immediate postglacial warming period, so that the present-day R0a frequency in parts of the Horn approaches 20%. This supports the pre-agricultural gene flow recently inferred from genome-wide data^10^, and may be linked to the establishment of obsidian exchange networks across the Red Sea in the early Holocene^64^,^65^. Both sets of analyses contrast with the previously established scenario that most of the non-African ancestry in the Horn is the result of admixture ~3 ka^11^,^12^. However, Hodgson *et al.*^10^ argue cogently that genome-wide dating methods based on linkage disequilibrium are strongly biased in favour of recent admixture events (see also^15^), and propose a deep Pleistocene ancestry for the Eurasian admixture, dating back as much as 23 ka. On the other hand, they and others^11^ also propose that the Eurasian admixture in the Horn came from the northeast, rather than from Arabia.

However, the limitations to current genome-wide analyses extend beyond the timing of dispersals to the identification of source populations, which can often be clarified on the basis of the phylogenetic nesting relationships identifiable with the non-recombining marker systems. In fact, the mtDNA evidence clearly indicates that Eurasian admixture in the Horn indeed occurred several times, and from several distinct sources. In addition to R0a, there are four other potentially Eurasian ancient mtDNA clades in Eastern Africa: M1a, U6a, HV1 and N1a1a, which together with R0a make up 30% of Ethiopian lineages in our control-region database (*n* = 169). There is also a smattering of “accidental” lineages (7%) that most likely arrived within the last few centuries, so about 81% of the Eurasian lineages in Ethiopia are potentially ancient.

However, aside from R0a, only one other haplogroup is likely to indicate a Pleistocene dispersal from Arabia: N1a1a^3^. N1a1a3 dates to ~15.2 ka and N1a1a4 to only 850 years, but both diverge directly from the N1a1a root, which dates to ~25 ka, with the only closely related lineages seen in Arabia – a clearly similar pattern to R0a. HV1b1 in the Horn also has a Yemen source and dates to ~8.2 ka, leading to the suggestion of an early Holocene movement^18^, but it is interleaved with Yemeni lineages in the tree, suggesting that it may have arrived more recently. A very approximate founder age estimate suggests an arrival ~5 ka.

More frequent even than R0a in the Horn is M1a, thought to have arrived during the Late Glacial^16^. There are few lineages from which to estimate an arrival time, but M1a1c’d dates to ~12.0 ka. However, M1a probably arrived via Egypt rather than Yemen^44^. Another North African/Mediterranean lineage, haplogroup U6a, again has a likely source in Egypt/Near East^44^,^66^, but U6a2a1 in the Horn dates to ~4.0 ka.

In summary there were several late Pleistocene arrivals, from both North Africa/Levant and from Arabia, and similarly there seem likely to have been several mid-Holocene arrivals, again from both sources. Overall, about 62% of the Eurasian lineages probably arrived in Ethiopia during the Pleistocene (~33% from Arabia and ~29% from the north), with ~19% in the mid-Holocene (but half from Arabia and half from the north), with the remaining ~19% likely very recent. Potentially, all of these different ages are conflated into the autosomal admixture estimate of 3 ka.

Our results do indicate population growth within Arabia at ~3 ka, which may be implicated in a late Holocene range expansion across the Arabian Sea involving perhaps HV1, and perhaps also of R0a1a1a lineages into the island of Socotra, where the age of the R0a1a1 lineages date to the same timeframe^35^. Populations survived along the southeast Arabian coast during the extreme aridity of the so-called “Dark Millennium” after 5.9 ka and may have prospered as climatic conditions improved again in the Arabian Bronze Age. Although there is less evidence from Yemen, this phase saw marked re-settlement of southeast Arabia during the Hafit phase of oasis agriculture after 5.1 ka^67^, and a similar trajectory seems likely to have taken place to the west.

The return to more pluvial conditions in Eastern Africa appears to have been later, ~4 ka^68^, matching estimates for the establishment of Ethiosemitic languages in the Horn^14^. It also coincides with the appearance of the poorly-known literate *Daamat-Di’amat* polity in northern Ethiopia/Eritrea, which extended from roughly 850–350 BC, and has long been thought to show signs of Arabian influence^69^. However, some recent archaeological studies have downplayed the extent of Arabian influence and consider large-scale migration at this time unlikely, more in line with the evidence that we present here^70^. There may have been some minor gene flow due to the intensification of maritime trading networks that had begun around this time^34^,^69^, also indicated by the appearance of R0a lineage around the Indian Ocean as far as India. But the main episodes of Arabian settlement in the Horn occurred much earlier, at the end of the Ice Age.

## Subjects and Methods

We identified candidate R0a mtDNAs by surveying control-region mutational motifs of ~10,000 subjects of various geographic origins (Africa, East and South Asia, the Near East, Caucasus and Europe) whose DNA was available in the laboratories participating in this study. For all subjects, we obtained appropriate written informed consent, and the research was reviewed and approved by the Ethics Committee for Clinical Experimentation of the University of Pavia (Italy), board minutes of April 11^th^ 2013. All experiments were performed in accordance with relevant guidelines and regulations.

We PCR-amplified 54 candidate R0a mtDNAs, selected on the basis of the presence of the diagnostic R0a control-region motif, and completely sequenced them following a well-established Sanger protocol^71^. We aligned, assembled, and compared them using Sequencher 5.0 (Gene Codes Corporation) relative to both the Revised Sapiens Reference Sequence (RSRS)^33^ and the revised Cambridge Reference Sequence (rCRS)^72^. We also identified and sequenced two candidate R0b mtDNAs, following the same approach.

We furthermore sequenced five additional candidate R0a mtDNAs (from the Csangos and Szekelys of Romania, see^73^ for sampling) using massively parallel sequencing. We enriched these mitogenomes in 62 midi-sized amplicons, purified them using AMPure XP beads (Beckman Coulter, Inc., Brea, CA, USA) and obtained MiSeq libraries using the Nextera XT DNA Sample preparation kit^74^. We performed MPS using the Illumina MiSeq benchtop sequencer (Illumina, San Diego, CA, USA) following the manufacturer’s recommendations. We analysed MiSeq-generated sequences using the NextGENe software (SoftGenetics, State College, PA, USA) and assessed them relative to both the internal MiSeq Reporter results and the corresponding control-region sequences^73^. We clarified remaining inconsistencies by Sanger sequencing to forensic quality standards^75^.

We analysed the 61 novel mitogenomes alongside 143 R0a (and one R0b) whole-mtDNA sequences already available in public databases. Geographic and/or ethnic affiliations of the 202 R0a mtDNAs, as well as their accession numbers are listed in Table S1, together with the two novel and one previously reported^76^ R0b mitogenomes. For the construction of the R0a phylogeny we employed a maximum parsimony approach with the aid of the mtPhyl software (http://eltsov.org/mtphyl.aspx), correcting the tree by hand with reference to PhyloTree (Build 17)^77^. A new haplogroup label was assigned following the established nomenclature only when the candidate haplogroup encompassed at least two haplotypes. We disregarded the unreliable np 60 when identifying clades.

We estimated coalescence times using both maximum likelihood (ML) and the ρ statistic (average distance of the haplotypes of a clade from the respective root haplotype)^78^ accompanied by a heuristic estimate of the standard error (σ) calculated from an estimate of the genealogy^79^. We used PAML 4.5^80^ to obtain ML estimates, assuming the HKY85 mutation model (two parameters in the model of DNA evolution) with γ -distributed rates (approximated by a discrete distribution with 32 categories) and two partitions: coding region (from np 577 to np 16023) and control region (from np 16024 to np 576). We performed these calculations considering all substitutions except those at np 16519 and the 16182C and 16183C. We converted mutational distances into years using the substitution rate of about one mutation every 3,624 years for the entire mitogenome, and correcting for purifying selection using the calculator provided by Soares *et al.*^81^.

We also obtained Bayesian skyline plots (BSPs)^82^ from BEAST 1.7.4^83^ for haplogroup R0a and its most frequent subclades, using a strict molecular clock (lognormal distribution across branches and uncorrelated between them) and a HKY85-type model with γ-distributed rates. BSPs estimate effective population size through time from random sequences of a population. Haplogroups in general do not equate to population data, but the signal associated with a haplogroup might nevertheless signal demographic processes in the populations carrying it, as previously suggested^52^. To approximate the mutation rate to the one used in previous analyses, we used a U6 sequence (EF064317) as an outgroup^44^, setting the age of haplogroup R to ~59 ka (95% C.I: 49–69 ka), as an average of previously proposed estimates^33^,^81^; plus we considered the age obtained here for R0a as consistent internal calibration points^52^. Specifically, we ran 50,000,000 iterations, with samples drawn every 10,000 Markov chain Monte Carlo (MCMC) steps, after a discarded burn-in of 10,000,000 steps, as in Soares *et al.*^52^. We considered haplogroup R0a and its major subclades monophyletic in the analyses. We visualized the BSPs obtained in plots with Tracer v1.5 and Excel using a generation time of 25 years.

In order to estimate the times of migrations, we employed founder analysis^30^. This method assumes a strict division between assumed source and sink populations and two criteria (*f1* and *f2*) for identifying founder sequences to allow as far as possible for homoplasy and back-migrations, by ensuring that sequence matches are not at the tips of the source phylogeny. We thus stipulate that founders must have at least one (*f1*) or two (*f2*) derived branches in the source population. We carried out the analysis for R0a using the whole mitogenomes – estimating the age of the migration of each founder using the ρ statistic. However, since the assumptions of the founder method do not allow the use of a time-dependent clock, as usually performed for whole mitogenomes^81^, we used an approximated linear rate. Given the relatively small difference between the mutation rate for time zero (average 2562 years for a mutation to happen) and the mutation rate for the oldest estimated founder (average 2667 years for a mutation to happen) we used the intermediate value between these (2651 years for a mutation to happen) as an estimate for the overall range, as previously^15^. We performed the founder analysis in several ways to estimate the arrival times of R0a lineages in different continents, in each case performing the analyses with Palestinians included either with Arabian populations, with Fertile Crescent populations or unassigned, and also either including and excluding Iranian lineages from the Fertile Crescent source. We performed the following analyses: (1) from the Near East into Arabia/Eastern Africa; (2) Near East into Arabia; (3) Near East/Arabia into Eastern Africa; (4) Arabia into the Fertile Crescent (a “reciprocal” founder analysis to check the direction of dispersal: see^84^; (5) Near East/Arabia into South Asia; (6) Near East/Arabia into Europe. We scanned the distribution of founder ages for each region, defining equally spaced 200-year intervals for each migration from 0 to 50 ka.

We further assessed extant frequencies and geographical distributions of R0a and R0a1a by surveying published and unpublished datasets (more than 45,000 control-region sequences) for their diagnostic control-region mutational motifs. By searching our in-house database of unpublished sequences, the European DNA Profiling Group Mitochondrial Population Database (EMPOP)^85^,^86^, and published control-region sequences (mainly limited to HVS-I), we were able to evaluate a total of more than 100 populations (Table S2). We constructed spatial frequency distribution plots with the program Surfer 9 (Golden Software). We extended the search to members of two of the largest subclades restricted mainly to Eastern Africa, R0a2b1 and R0a2b2. Unfortunately, R0a2g, a third essentially African-specific subclade, does not harbour any diagnostic mutation in the control region, so that its geographic distribution could not be further evaluated. We also constructed a phylogenetic network of the HVS-I variation in R0a’b using the Network package (Fig. S1).

## Additional Information

**How to cite this article**: Gandini, F. *et al.* Mapping human dispersals into the Horn of Africa from Arabian Ice Age refugia using mitogenomes. *Sci. Rep.* **6**, 25472; doi: 10.1038/srep25472 (2016).

## Supplementary Material

Supplementary Information

## Figures and Tables

**Table 1:** Molecular divergence and age estimates (maximum likelihood and ρ) for haplogroup R0a’b and its subclades.

Haplogroup	N^a^	ML^b^	SE	Age (ka)^c^	95% CI (ka)	ρ	σ	Age (ka)^c^	95% CI (ka)
R0a’b	205	13.77	1.20	39.20	(32.01;46.57)	10.85	2.02	30.30	(23.97;36.79)
>R0a	202	10.49	0.79	29.22	(24.64;33.89)	7.82	1.09	21.37	(15.28;27.63)
>>R0a1	58	9.54	0.87	26.39	(21.43;31.45)	8.00	1.85	21.89	(11.64;32.64)
>>>R0a1a	52	4.81	0.36	12.85	(10.92;14.80)	4.13	0.46	10.97	(8.51;13.46)
>>>>R0a1a1	12	3.89	0.56	10.31	(7.31;13.36)	2.67	0.93	7.00	(2.19;11.97)
>>>>>R0a1a1a	9	1.35	0.38	3.51	(1.59;5.46)	1.22	0.62	3.16	(0.01;6.38)
>>>>>>R0a1a1a1	5	0.63	0.26	1.63	(0.30;2.98)	0.40	0.28	1.03	(0;2.47)
>>>>R0a1a2	3	1.79	0.45	4.65	(2.33;7.01)	2.33	1.00	6.09	(0.95;11.41)
>>>>R0a1a3	4	4.41	0.41	11.73	(9.51;13.98)	6.25	1.75	16.88	(7.41;26.81)
>>>>>R0a1a3a	3	2.16	0.64	5.64	(2.34;9.01)	2.67	1.25	7.00	(0.56;13.70)
>>>>R0a1a4	3	2.85	0.64	7.49	(4.16;10.90)	3.33	1.25	8.78	(2.27;15.55)
>>>>R0a1a5	4	3.49	0.60	9.21	(6.03;12.45)	2.75	0.83	7.22	(2.91;11.65)
>>>R0a1b	3	0.69	3.67	1.78	(0;21.55)	0.67	1.54	1.73	(0;9.76)
>>R0a2’3	123	7.66	0.83	20.92	(16.29;25.65)	6.06	1.06	16.34	(10.56;22.29)
>>>R0a2	117	6.14	0.41	16.56	(14.28;18.87)	5.10	0.49	13.65	(10.99;16.34)
>>>>R0a2a	7	3.34	0.88	8.82	(4.18;13.58)	2.29	0.70	5.99	(2.37;9.69)
>>>>>R0a2a1	3	0.98	0.43	2.53	(0.34;4.76)	0.67	0.47	1.73	(0;4.14)
>>>>R0a2b	24	4.75	0.64	12.69	(9.25;16.19)	4.29	1.16	11.41	(5.26;17.77)
>>>>>R0a2b1	14	4.01	0.66	10.62	(7.12;14.20)	4.21	1.41	11.19	(3.75;18.94)
>>>>>>R0a2b1a	9	0.26	0.11	0.67	(0.10;1.24)	0.22	0.16	0.56	(0;1.37)
>>>>>>R0a2b1b	5	3.05	0.66	8.04	(4.59;11.56)	3.20	1.26	8.43	(1.88;15.24)
>>>>>>>R0a2b1b1	4	1.76	0.43	4.57	(2.35;6.83)	1.75	0.66	4.55	(1.17;8.01)
>>>>>R0a2b2	9	1.50	0.49	3.89	(1.39;6.43)	1.11	0.47	2.87	(0.48;5.30)
>>>>R0a2c	4	4.64	1.05	12.37	(6.75;18.17)	3.50	1.41	9.25	(1.90;16.92)
>>>>>R0a2c1	3	0.84	0.41	2.16	(0.06;4.28)	0.67	0.47	1.73	(0;4.14)
>>>>R0a2d	7	4.67	0.81	12.45	(8.11;16.89)	4.43	1.17	11.79	(5.58;18.22)
>>>>R0a2f	15	5.56	0.53	14.94	(12.06;17.85)	6.27	1.58	16.93	(8.36;25.89)
>>>>>R0a2f1	9	2.07	0.53	5.41	(2.70;8.18)	2.22	0.93	5.80	(1.02;10.73)
>>>>>>R0a2f1a	6	1.07	0.45	2.76	(0.47;5.09)	1.17	0.73	3.03	(0;6.82)
>>>>>>R0a2f1b	3	1.27	0.49	3.29	(0.79;5.82)	1.33	0.82	3.45	(0;7.72)
>>>>R0a2g	7	4.15	0.94	11.02	(6.03;16.15)	3.29	0.96	8.68	(3.65;13.85)
>>>>>R0a2g1	4	3.14	0.71	8.27	(4.53;12.10)	3.00	1.12	7.89	(2.08;13.91)
>>>>>>R0a2g1a	3	2.48	0.64	6.49	(3.17;9.88)	2.67	1.05	7.00	(1.58;12.62)
>>>>R0a2h	3	3.49	0.92	9.21	(4.37;14.19)	2.67	1.15	7.00	(1.07;13.16)
>>>>R0a2i	3	1.64	0.64	4.27	(1.00;7.61)	1.67	0.88	4.34	(0;8.96)
>>>>>R0a2j	3	5.16	0.68	13.81	(10.15;17.55)	6.67	1.56	18.07	(9.55;26.95)
>>>>R0a2k	3	5.50	0.53	14.78	(11.91;17.69)	7.00	1.97	19.01	(8.27;30.33)
>>>>R0a2m	3	0.55	0.36	1.41	(0;3.23)	0.50	0.50	1.29	(0;3.84)
>>>>R0a2n	7	4.93	0.83	13.17	(8.72;17.73)	3.86	1.19	10.23	(3.97;16.72)
>>>>>R0a2n1	4	2.45	1.22	6.41	(0.13;12.94)	1.75	1.09	4.55	(0;10.30)
>>>>>R0a2n2	3	2.45	0.70	6.41	(2.81;10.10)	2.33	0.88	6.09	(1.56;10.76)
>>>>R0a2o	5	3.83	0.90	10.15	(5.38;15.05)	2.40	1.02	6.28	(1.03;11.71)
>>>>>R0a2o1	4	1.67	0.62	4.35	(1.17;7.59)	1.25	0.66	3.24	(0;6.67)
>>>>R0a2q	4	1.47	0.64	3.82	(0.55;7.15)	1.00	0.61	2.59	(0;5.74)
>>>>R0a2r	11	4.64	0.85	12.37	(7.84;17.02)	3.82	1.20	10.12	(3.81;16.66)
>>>R0a3	5	5.19	0.87	13.89	(9.22;18.68)	3.80	1.08	10.06	(4.38;15.94)
>>>>R0a3a	3	4.24	0.85	11.26	(6.75;15.88)	3.67	1.29	9.71	(2.96;16.73)
>>R0a4	6	0.46	0.21	1.19	(0.14;2.24)	0.33	0.24	0.85	(0;2.07)
>>R0a5	2	6.92	1.19	18.77	(12.24;25.51)	5.50	1.66	14.77	(5.87;24.09)
>>R0a6	11	1.15	0.38	2.98	(1.07;4.92)	1.00	0.35	2.59	(0.81;4.39)
>R0b	3	5.71	1.00	15.34	(9.93;20.91)	5.00	1.37	13.37	(6.05;20.98)

^a^Number of mitogenomes.

^b^Maximum likelihood molecular divergence.

^c^Using the corrected molecular clock proposed by Soares *et al.*^81^. Except for R0a5, we calculated age estimates only for subclades encompassing at least three mitogenomes.
