# Supplementary material for: Mapping human dispersals into the Horn of Africa from Arabian Ice Age refugia using mitogenomes
Source: Sci Rep. 2016 May 5;6:25472. doi: 10.1038/srep25472 (PMC4857117; doi:10.1038/srep25472)
Supplement: Supplementary Information [file srep25472-s1.pdf]

SUPPLEMENTARY INFORMATION

Mapping human dispersals into the Horn of Africa from Arabian Ice Age refugia using mitogenomes

Francesca Gandini[1,2], Alessandro Achilli[1,3], Maria Pala[2], Martin Bodner[4], Stefania Brandini[1], Gabriela Huber[4], Balazs Egyed[5], Luca Ferretti[1], Alberto Gómez-Carballa[6], Antonio Salas[6], Rosaria Scozzari[7], Fulvio Cruciani[7], Alfredo Coppa[8], Walther Parson[4,9], Ornella Semino[1], Pedro Soares[10], Antonio Torroni[1], Martin B. Richards[2*], Anna Olivieri[1*]

[1]Dipartimento di Biologia e Biotecnologie "L. Spallanzani", Università di Pavia, Pavia, Italy;

[2]School of Applied Sciences, University of Huddersfield, Queensgate, Huddersfield, UK;

[3]Dipartimento di Chimica, Biologia e Biotecnologie, Università di Perugia, Perugia, Italy;

[4]Institute of Legal Medicine, Medical University of Innsbruck, Innsbruck, Austria;

[5]Department of Genetics, Eötvös Loránd University, Budapest, Hungary;

[6]Unidade de Xenética, Departamento de Anatomía Patolóxica e Ciencias Forenses, and Instituto de Ciencias Forenses, Facultade de Medicina, Universidad de Santiago de Compostela, Santiago de Compostela 15782, Galicia, Spain;

[7]Dipartimento di Biologia e Biotecnologie "Charles Darwin", Sapienza Università di Roma, Rome, Italy;

[8]Dipartimento di Biologia Ambientale, Sapienza Università di Roma, Rome, Italy;

[9]Forensic Science Program, The Pennsylvania State University, University Park, Pennsylvania, USA;

[10]CBMA (Centre of Molecular and Environmental Biology), Department of Biology, University of Minho, Campus de Gualtar, 4710-057 Braga, Portugal.

[*]E-mails: m.b.richards@hud.ac.uk; anna.olivieri@unipv.it

Table S1. Origin and sub-haplogroup affiliation of the mitogenomes analysed in this study.

ID #	Accession number [a]	Haplogroup	Geographic region [b]	Country and/or ethnicity [b]	Reference
**1**	KF451216	R0a1a	Fertile Crescent	Israel (Central, Palestinian)	[1]
**2**	KJ446219	R0a1a	Fertile Crescent	Israel (Central, Palestinian)	[2]
**3**	HM185210	R0a1a1a	Arabian Peninsula	Yemen (Socotra)	[3]
**4**	HM185211	R0a1a1a	Arabian Peninsula	Yemen (Socotra)	[3]
**5**	HM185230	R0a1a1a	Arabian Peninsula	Yemen (Al Mahra)	[3]
**6**	HM185208	R0a1a1a1	Arabian Peninsula	Yemen (Socotra)	[3]
**7**	HM185209	R0a1a1a1	Arabian Peninsula	Yemen (Socotra)	[3]
**8**	HM185207	R0a1a1a1	Arabian Peninsula	Yemen (Socotra)	[3]
**9**	HM185206	R0a1a1a1	Arabian Peninsula	Yemen (Socotra)	[3]
**10**	HM185236	R0a1a1a1	Arabian Peninsula	Yemen (Socotra)	[3]
**11**	HM185205	R0a1a1a	Arabian Peninsula	Yemen (Socotra)	[3]
**12**	HM185217	R0a1a1	Arabian Peninsula	Yemen (Socotra)	[3]
**13**	HM185263	R0a1a1	North Africa	Tunisia	[3]
**14**	KP407022	R0a1a1	Arabian Peninsula	Yemen	This study
**15**	HM185260	R0a1a2a	Eastern Africa	Ethiopia	[3]
**16**	HM185258	R0a1a2a	Eastern Africa	Ethiopia	[3]
**17**	KP407023	R0a1a2	Eastern Africa	Ethiopia (Tigray)	This study
**18**	HM185213	R0a1a3a1	Arabian Peninsula	Yemen	[3]
**19**	HM185212	R0a1a3a1	Arabian Peninsula	Yemen	[3]
**20**	GU592021	R0a1a3a	Western Europe	Austria	[4]
**21**	KC911515	R0a1a3	Iran	Iran	[5]
**22**	HM185247	R0a1a4a	Arabian Peninsula	Yemen	[3]
**23**	HM185245	R0a1a4a	Arabian Peninsula	Yemen	[3]
**24**	HM185233	R0a1a4	Arabian Peninsula	Yemen (Al Mahra)	[3]
**25**	KP407028	R0a1a5	Fertile Crescent	Palestinian	This study
**26**	HM185232	R0a1a5	Arabian Peninsula	Yemen (Al Mahra)	[3]
**27**	KC911447	R0a1a5	Iran	Iran	[5]
**28**	DQ904237	R0a1a5	Arabian Peninsula	Saudi Arabia	[6]
**29**	KP407029	R0a1a6	Western Europe	Italy (Campania)	This study
**30**	HM185268	R0a1a6	Eastern Africa	Somalia	[3]
**31**	KP407030	R0a1a7	Fertile Crescent	Syria	This study
**32**	JX153020	R0a1a7	Western Europe	Italy	[7]
**33**	KF451215	R0a1a8	Fertile Crescent	Israel (Central, Palestinian)	[1]
**34**	KC911389	R0a1a8	Iran	Iran	[5]
**35**	KP407024	R0a1a	Eastern Africa	Ethiopia (Afar)	This study
**36**	HM185216	R0a1a	Arabian Peninsula	Yemen	[3]
**37**	HM185262	R0a1a	North Africa	Tunisia	[3]
**38**	JQ705369	R0a1a	NA	NA	[8]
**39**	HM185252	R0a1a	Arabian Peninsula	Yemen	[3]
**40**	KP407026	R0a1a	Fertile Crescent	Palestinian	This study
**41**	HM185203	R0a1a	Arabian Peninsula	Yemen	[3]
**42**	KP407027	R0a1a	Arabian Peninsula	Yemen	This study
**43**	HM185251	R0a1a	Arabian Peninsula	Yemen	[3]
**44**	DQ904235	R0a1a	Arabian Peninsula	Saudi Arabia	[6]
**45**	FJ460524	R0a1a	North Africa	Tunisia	[9]
**46**	HM185204	R0a1a	Arabian Peninsula	Yemen	[3]
**47**	DQ904239	R0a1a	Arabian Peninsula	Saudi Arabia	[6]
**48**	KF451206	R0a1a	Fertile Crescent	Israel (Central, Palestinian)	[1]
**49**	KF451213	R0a1a	Fertile Crescent	Israel (Central, Palestinian)	[1]
**50**	EF660971	R0a1a	Western Europe	Italy	[10]
**51**	KP407031	R0a1a	Arabian Peninsula	Yemen	This study
**52**	KP407025	R0a1a	Arabian Peninsula	Yemen	This study
**53**	DQ904236	R0a1b	Arabian Peninsula	Saudi Arabia	[6]

**54**	HM185240	R0a1b	Arabian Peninsula	Yemen	[3]
**55**	HM185272	R0a1b	Eastern Africa	Sudan	[3]
**56**	KF451143	R0a1	Fertile Crescent	Israel (Negev, Bedouin)	[1]
**57**	HM185256	R0a1	North Africa	Morocco	[3]
**58**	JQ702678	R0a1	NA	NA	[8]
**59**	HM185257	R0a2a1	North Africa	Morocco	[3]
**60**	JQ702181	R0a2a1	Western Europe	Italy	[8]
**61**	HM185264	R0a2a1	North Africa	Tunisia	[3]
**62**	EF556170	R0a2a	North Africa	Tunisia	[11]
**63**	KP407032	R0a2a	Western Europe	Spain (Sevilla)	This study
**64**	HM185270	R0a2a	Eastern Africa	Sudan	[3]
**65**	KP407033	R0a2a	Western Europe	Spain (Murcia)	This study
**66**	KP407034	R0a2b1a	Eastern Africa	Eritrea (Saho)	This study
**67**	KP407035	R0a2b1a	Eastern Africa	Eritrea (Saho)	This study
**68**	KP407036	R0a2b1a	Eastern Africa	Eritrea (Saho)	This study
**69**	KP407037	R0a2b1a	Eastern Africa	Eritrea (Saho)	This study
**70**	KP407038	R0a2b1a	Eastern Africa	Eritrea (Saho)	This study
**71**	KP407039	R0a2b1a	Eastern Africa	Eritrea (Saho)	This study
**72**	KP407040	R0a2b1a	Eastern Africa	Eritrea (Saho)	This study
**73**	KP407041	R0a2b1a	Eastern Africa	Ethiopia (Oromo)	This study
**74**	KP407042	R0a2b1a	Eastern Africa	Eritrea (Saho)	This study
**75**	KP407044	R0a2b1b1	Eastern Africa	Ethiopia (Oromo)	This study
**76**	KP407045	R0a2b1b1	Eastern Africa	Ethiopia (Gurage)	This study
**77**	HM185249	R0a2b1b1	Arabian Peninsula	Yemen	[3]
**78**	KP407046	R0a2b1b1	Arabian Peninsula	Yemen	This study
**79**	KP407043	R0a2b1b	Eastern Africa	Kenya (Oromo)	This study
**80**	KP407048	R0a2b2	Eastern Africa	Ethiopia (Oromo)	This study
**81**	KP407049	R0a2b2	Eastern Africa	Ethiopia (Afar)	This study
**82**	KP407050	R0a2b2	Eastern Africa	Ethiopia (Afar)	This study
**83**	KP407051	R0a2b2	Fertile Crescent	Palestinian (Gaza)	This study
**84**	HM185255	R0a2b2	Eastern Africa	Ethiopia (Jew)	[3]
**85**	EF556172	R0a2b2	Eastern Africa	Ethiopia (Beta Israel)	[11]
**86**	HM185261	R0a2b2	Eastern Africa	Ethiopia	[3]
**87**	KP407052	R0a2b2	Eastern Africa	Eritrea (Afar)	This study
**88**	KP407053	R0a2b2	Eastern Africa	Eritrea (Saho)	This study
**89**	KP407047	R0a2b	Fertile Crescent	Palestinian	This study
**90**	KP407054	R0a2c1	Arabian Peninsula	Yemen	This study
**91**	EF556176	R0a2c1	Arabian Peninsula	Yemen	[11]
**92**	KP407055	R0a2c1	Arabian Peninsula	Yemen	This study
**93**	DQ904238	R0a2c	Arabian Peninsula	Saudi Arabia	[6]
**94**	JF717359	R0a2d	Western Europe	Italy	[12]
**95**	JF717360	R0a2d	Western Europe	Italy	[12]
**96**	EF436244	R0a2d	NA	NA	[13]
**97**	AY738940	R0a2d	Central and South Asia	Pakistan	[14]
**98**	KF450946	R0a2d	Central and South Asia	Pakistan (Pathan)	[1]
**99**	HM185241	R0a2d	Arabian Peninsula	Yemen	[3]
**100**	KC911494	R0a2d	Iran	Iran	[5]
**101**	DQ904242	R0a2e	Western Europe	Iberia	[6]
**102**	HM185227	R0a2f1a	Arabian Peninsula	Yemen (Al Mahra)	[3]
**103**	HM185219	R0a2f1a	Arabian Peninsula	Yemen (Socotra)	[3]
**104**	HM185221	R0a2f1a	Arabian Peninsula	Yemen (Socotra)	[3]
**105**	HM185222	R0a2f1a	Arabian Peninsula	Yemen (Socotra)	[3]
**106**	HM185234	R0a2f1a	Arabian Peninsula	Yemen (Socotra)	[3]
**107**	HM185220	R0a2f1a	Arabian Peninsula	Yemen (Socotra)	[3]
**108**	HM185214	R0a2f1b1	Arabian Peninsula	Yemen	[3]
**109**	HM185228	R0a2f1b1	Arabian Peninsula	Yemen (Al Mahra)	[3]
**110**	HM185248	R0a2f1b1	Arabian Peninsula	Yemen (Al Mahra)	[3]

**111**	EF660974	R0a2f	Western Europe	Italy	[10]
**112**	HM185225	R0a2f	North Africa	Chad	[3]
**113**	HM185226	R0a2f	North Africa	Chad	[3]
**114**	KF451176	R0a2f	Fertile Crescent	Israel (Negev, Bedouin)	[1]
**115**	KF451159	R0a2f	Fertile Crescent	Israel (Negev, Bedouin)	[1]
**116**	KP407056	R0a2f	Arabian Peninsula	United Arab Emirates (Dubai)	This study
**117**	KP407059	R0a2g1a1	Eastern Africa	Ethiopia (Amhara)	This study
**118**	KP407060	R0a2g1a1	Eastern Africa	Eritrea (Saho)	This study
**119**	KP407058	R0a2g1a	Eastern Africa	Eritrea (Afar)	This study
**120**	KP407057	R0a2g1	Eastern Africa	Ethiopia (Oromo)	This study
**121**	HM185218	R0a2g	Arabian Peninsula	Yemen	[3]
**122**	HM185271	R0a2g	Eastern Africa	Sudan	[3]
**123**	HM185269	R0a2g	Eastern Africa	Somalia	[3]
**124**	KP407061	R0a2h1	Eastern Africa	Eritrea (Afar)	This study
**125**	HM185238	R0a2h1	Arabian Peninsula	Yemen	[3]
**126**	DQ904240	R0a2h	Arabian Peninsula	Saudi Arabia	[6]
**127**	HM185243	R0a2i1	Arabian Peninsula	Yemen	[3]
**128**	HM185242	R0a2i1	Arabian Peninsula	Yemen	[3]
**129**	DQ904241	R0a2i	Arabian Peninsula	Saudi Arabia	[6]
**130**	HM185229	R0a2j	Arabian Peninsula	Yemen (Al Mahra)	[3]
**131**	HM185231	R0a2j	Arabian Peninsula	Yemen (Al Mahra)	[3]
**132**	HM185246	R0a2j	Arabian Peninsula	Yemen	[3]
**133**	HM185237	R0a2	Arabian Peninsula	Yemen	[3]
**134**	HM185254	R0a2k1	Arabian Peninsula	Yemen	[3]
**135**	HM185253	R0a2k1	Arabian Peninsula	Yemen	[3]
**136**	JF717355	R0a2k	Western Europe	Italy	[12]
**137**	HM185250	R0a2l	Arabian Peninsula	Yemen	[3]
**138**	HM185244	R0a2l	Arabian Peninsula	Yemen	[3]
**139**	JQ705916	R0a2m	NA	NA	[8]
**140**	JQ705196	R0a2m	Eastern Europe	Ukraine (Ashkenazi)	[8]
**141**	JQ703505	R0a2m	Eastern Europe	Poland	[8]
**142**	HM185215	R0a2o	Arabian Peninsula	Yemen	[3]
**143**	KF451139	R0a2o1	Fertile Crescent	Israel (Negev, Bedouin)	[1]
**144**	KF451156	R0a2o1	Fertile Crescent	Israel (Negev, Bedouin)	[1]
**145**	KF451151	R0a2o1	Fertile Crescent	Israel (Negev, Bedouin)	[1]
**146**	KP407062	R0a2o1	Fertile Crescent	Lebanon (Druze)	This study
**147**	JF717356	R0a2n1	Western Europe	Italy	[12]
**148**	JF717357	R0a2n1	Western Europe	Italy	[12]
**149**	JF717358	R0a2n1	Western Europe	Italy	[12]
**150**	KC911373	R0a2n1	Iran	Iran	[5]
**151**	KM103654	R0a2n2	Eastern Europe	Croat from Bosnia and Herzegovina	[15]
**152**	HM185259	R0a2n2	Eastern Africa	Ethiopia	[3]
**153**	HM185235	R0a2n2	Arabian Peninsula	Yemen (Socotra)	[3]
**154**	HG02657.PJL	R0a2p	Central and South Asia	Pakistan	[16]
**155**	AY713999	R0a2p	Central and South Asia	India	[17]
**156**	KP407063	R0a2q	Eastern Africa	Kenya (Oromo)	This study
**157**	KP407064	R0a2q	Eastern Africa	Eritrea (Saho)	This study
**158**	KP407065	R0a2q	Eastern Africa	Eritrea (Saho)	This study
**159**	HM185266	R0a2q	Eastern Africa	Somalia	[3]
**160**	KP407066	R0a2r	Fertile Crescent	Lebanon (Druze)	This study
**161**	KP407069	R0a2r	Eastern Europe	Bulgaria	This study
**162**	KP407070	R0a2r	Eastern Europe	Romania (Szekler) [c]	This study
**163**	KP407071	R0a2r	Eastern Europe	Romania (Szekler) [c]	This study
**164**	KP407068	R0a2r	Eastern Europe	Romania (Csango) [c]	This study
**165**	KP407073	R0a2r	Eastern Europe	Romania (Csango) [c]	This study

**166**	KM103659	R0a2r	Eastern Europe	Macedonian	[15]
**167**	KP407072	R0a2r	Eastern Europe	Romania (Csango) [c]	This study
**168**	KP407067	R0a2r	Western Europe	Italy	This study
**169**	KJ446223	R0a2r	Fertile Crescent	Israel (Carmel, Druze)	[2]
**170**	JX297187	R0a2r	Western Europe	Basque Country	[18]
**171**	HM185273	R0a2	Eastern Africa	Sudan	[3]
**172**	KP407074	R0a2	Eastern Africa	Ethiopia (Afar)	This study
**173**	HM185267	R0a2	Eastern Africa	Somalia	[3]
**174**	HM185239	R0a2	Arabian Peninsula	Yemen	[3]
**175**	KF451201	R0a2	Fertile Crescent	Israel (Central, Palestinian)	[1]
**176**	KP407075	R0a3a	Arabian Peninsula	Yemen	This study
**177**	HM185224	R0a3a	Arabian Peninsula	Yemen	[3]
**178**	HM185265	R0a3a	North Africa	Tunisia	[3]
**179**	HM185223	R0a3	Arabian Peninsula	Yemen	[3]
**180**	KC911556	R0a3	Iran	Iran	[5]
**181**	HM852825	R0a2'3	Iran	Iran	[19]
**182**	JQ702940	R0a4	NA	NA	[8]
**183**	KP407076	R0a4	Western Europe	Spain (Córdoba)	This study
**184**	KP407077	R0a4	Western Europe	Spain (Malaga)	This study
**185**	HG01781.IBS	R0a4	Western Europe	Spain	[16]
**186**	KJ716336	R0a4	Fertile Crescent	Iraq (Baghdad)	[13]
**187**	JQ705305	R0a4	Western Europe	Germany	[8]
**188**	KP407078	R0a5	Western Europe	Spain (Salamanca)	This study
**189**	KP407079	R0a5	South Caucasus - Turkey	Turkey (Kurd)	This study
**190**	KF450972	R0a6	Central and South Asia	Pakistan (Kalash)	[1]
**191**	KF450974	R0a6	Central and South Asia	Pakistan (Kalash)	[1]
**192**	KF450982	R0a6	Central and South Asia	Pakistan (Kalash)	[1]
**193**	KF450967	R0a6	Central and South Asia	Pakistan (Kalash)	[1]
**194**	KF450966	R0a6	Central and South Asia	Pakistan (Kalash)	[1]
**195**	EU597493	R0a6	Central and South Asia	Pakistan	[20]
**196**	KF450968	R0a6	Central and South Asia	Pakistan (Kalash)	[1]
**197**	KF450986	R0a6	Central and South Asia	Pakistan (Kalash)	[1]
**198**	KP407080	R0a6	Fertile Crescent	Palestinian	This study
**199**	KF450980	R0a6	Central and South Asia	Pakistan (Kalash)	[1]
**200**	KJ446207	R0a6	Central and South Asia	Pakistan (Kalash)	[2]
**201**	KF055865	R0a	Western Europe	Spain (Romani)	[21]
**202**	JX153281	R0a	Western Europe	Italy	[7]
**203**	JF717361	R0b	Western Europe	Italy	[12]
**204**	KT272406	R0b1	South Caucasus - Turkey	Azerbaijan	This study
**205**	KT272407	R0b1	Western Europe	Italy	This study

[a] Accession numbers refer to GenBank except for #154 and #185 (1000 Genomes Project).

[b] NA = information not available.

[c] Hungarian ethnic group living in today Romania.

Table S2. Population frequencies (%) of haplogroup R0a and the subclades R0a1a, R0a2b1, R0a2b2 and R0a5.

Geographic Area	*Country/ Population*		*R0a*	*R0a1a*	*R0a2b1*	*R0a2b2*	*R0a5*	*n*	*References*
Asia			**0.484**	**0.039**	**0.000**	**0.000**	**0.013**	**7642**	
	Afghanistan		0.000	0.000	0.000	0.000	0.000	98	[22]
	China (west)		0.000	0.000	0.000	0.000	0.000	228	[23-26]
	India		0.187	0.000	0.000	0.000	0.000	2671	
		North-east	0.000	0.000	0.000	0.000	0.000	624	[27-30], Unpub
		North-west and North-centre	0.626	0.000	0.000	0.000	0.000	479	[27-29,31], Unpub
		Centre	0.000	0.000	0.000	0.000	0.000	131	[28-30]
		South-west	0.000	0.000	0.000	0.000	0.000	431	[27,29,32]
		South-east	0.199	0.000	0.000	0.000	0.000	1006	[27-30,33], Unpub
	Pakistan		2.120	0.092	0.000	0.000	0.000	1085	[28,31,34], Unpub
	Kazakhstan		0.477	0.000	0.000	0.000	0.239	419	[22-24,35]
	Kyrgyzstan		0.000	0.000	0.000	0.000	0.000	256	[22,35]
	Mongolia		0.000	0.000	0.000	0.000	0.000	199	[23,36,37]
	Nepal		0.000	0.000	0.000	0.000	0.000	168	Unpub
	Siberia		0.070	0.070	0.000	0.000	0.000	1436	
		West	0.000	0.000	0.000	0.000	0.000	313	[37,38]
		Centre	0.000	0.000	0.000	0.000	0.000	674	[37,38]
		East	0.223	0.223	0.000	0.000	0.000	449	[37-41]
	Uzbekistan		0.699	0.000	0.000	0.000	0.000	429	[22,23,31]
	Tajikistan		0.000	0.000	0.000	0.000	0.000	331	[22,31,37]
	Turkmen		0.932	0.311	0.000	0.000	0.000	322	[22,31]
Near East			**5.835**	**2.378**	**0.016**	**0.016**	**0.094**	**6392**	
	Bahrain		5.634	4.225	0.000	0.000	0.000	213	Unpub
	Iran		2.226	0.866	0.000	0.000	0.082	2426	
		North-east	2.492	0.623	0.000	0.000	0.312	321	[29,37], Unpub
		North-west	0.547	0.328	0.000	0.000	0.000	914	[29,31,37], Unpub
		Centre	3.817	1.527	0.000	0.000	0.000	655	[29,31], Unpub
		South	2.985	1.119	0.000	0.000	0.187	536	[29,31], Unpub
	Lebanon, Israel (Druze)		2.809	0.562	0.000	0.000	0.000	356	[42,43]
	Jordan		2.028	1.420	0.000	0.000	0.000	493	[44,45], Unpub
	Iraq		5.747	1.149	0.000	0.000	0.000	261	[44], Unpub
	Israel (Palestinians)		2.564	1.709	0.000	0.000	0.000	117	[44]
	Kuwait		12.132	3.125	0.000	0.000	0.000	544	[46], Unpub
	Turkey (Kurds)		1.220	0.000	0.000	0.000	1.220	82	[44,47]
	United Arab Emirates		5.221	2.410	0.000	0.000	0.803	249	[48]
	Yemen		14.286	7.418	0.275	0.000	0.000	364	[44,49-51], Unpub
	Saudi Arabia		17.526	6.701	0.000	0.172	0.000	582	[6,49]
	Soqotra Island		38.462	24.615	0.000	0.000	0.000	65	[52]
	Syria		3.390	1.695	0.000	0.000	0.000	118	[44,53]
	Turkey		1.149	0.192	0.000	0.000	0.192	522	[31,44,54], Unpub
Caucasus			**0.401**	**0.134**	**0.000**	**0.000**	**0.000**	**748**	
	Armenia		0.524	0.000	0.000	0.000	0.000	191	[44]
	Caucasus north /Chechnya/Ossetia/ Kabardian/ Kalmyk Republic		0.562	0.281	0.000	0.000	0.000	356	[31,37,44]
	Azerbaijan		0.000	0.000	0.000	0.000	0.000	88	[31,44]
	Georgia		0.000	0.000	0.000	0.000	0.000	113	[31,47,55]
Europe			**0.456**	**0.081**	**0.000**	**0.000**	**0.029**	**24561**	

Albania		0.000	0.000	0.000	0.000	0.000	42	[44]
Austria		0.267	0.000	0.000	0.000	0.000	374	[56,57]
Balearic Islands		5.078	0.000	0.000	0.000	0.000	256	[58,59]
Basque Country		0.000	0.000	0.000	0.000	0.000	321	[44,60-64]
Belgium		0.000	0.000	0.000	0.000	0.000	50	[65]
Bosnia-Herzegovina		1.389	0.000	0.000	0.000	0.000	144	[66]
Bulgaria		0.602	0.301	0.000	0.000	0.000	996	[44], Unpub
Corsica (south)		0.000	0.000	0.000	0.000	0.000	53	[59]
Croatia		0.000	0.000	0.000	0.000	0.000	96	[67]
Czech Republic		0.000	0.000	0.000	0.000	0.000	83	[44]
Denmark		0.000	0.000	0.000	0.000	0.000	244	[44]; [68]
England		0.000	0.000	0.000	0.000	0.000	345	[44,69]
Estonia		0.000	0.000	0.000	0.000	0.000	149	[44]
Finland		0.247	0.000	0.000	0.000	0.000	405	[44,70,71], Unpub
France		0.501	0.000	0.000	0.000	0.000	1198	
	North	0.333	0.000	0.000	0.000	0.000	600	[44,63,72]
	Centre	0.279	0.000	0.000	0.000	0.000	358	[44,63,73], Unpub
	South	1.250	0.000	0.000	0.000	0.000	240	[63,72]
Germany		0.141	0.000	0.000	0.000	0.000	1418	
	North	0.000	0.000	0.000	0.000	0.000	819	[74-79]
	South	0.334	0.000	0.000	0.000	0.000	599	[77,80,81], Unpub
Greece		1.209	0.605	0.000	0.000	0.000	827	[44,53,82], Unpub
Hungary		0.976	0.366	0.000	0.000	0.000	820	[83-85], Unpub
Iceland		0.000	0.000	0.000	0.000	0.000	457	[44,86]
Ireland		0.333	0.000	0.000	0.000	0.000	300	[87]
Italy		0.823	0.067	0.000	0.000	0.111	4498	
	Italy (general)	0.000	0.000	0.000	0.000	0.000	362	Unpub
	North	0.366	0.000	0.000	0.000	0.073	1366	[67,88,89], Unpub
	Centre	1.112	0.051	0.000	0.000	0.000	1979	[59,67,88,90,91], Unpub
	South	1.264	0.253	0.000	0.000	0.506	791	[44,67,92], Unpub
Latvia		0.000	0.000	0.000	0.000	0.000	299	[93]
Lithuania		0.000	0.000	0.000	0.000	0.000	180	[94]
Macedonia		1.002	0.601	0.000	0.000	0.000	499	[95], Unpub
Netherlands		0.962	0.000	0.000	0.000	0.000	104	Unpub
Poland		0.203	0.101	0.000	0.000	0.000	986	[44,96,97], Unpub
Portugal		0.629	0.070	0.000	0.000	0.140	1430	
	North	0.538	0.000	0.000	0.000	0.179	558	[64,98-100]
	Centre	0.542	0.000	0.000	0.000	0.181	553	[64,98-100]
	South	0.940	0.313	0.000	0.000	0.000	319	[98-100]
Romania		2.174	0.000	0.000	0.000	0.000	92	[44]
Russia (west)		0.120	0.000	0.000	0.000	0.000	835	[22,44,96,101-103]
Norway		0.000	0.000	0.000	0.000	0.000	628	[44,69,104]
Saami		0.000	0.000	0.000	0.000	0.000	294	[70,105-107], Unpub
Sardinia		0.000	0.000	0.000	0.000	0.000	1224	[44,59], Unpub
Scotland		0.000	0.000	0.000	0.000	0.000	1199	[69]
Serbia		0.000	0.000	0.000	0.000	0.000	104	[108]
Spain		0.197	0.049	0.000	0.000	0.000	2029	
	North	0.342	0.085	0.000	0.000	0.000	1170	[62,98,109-113], Unpub
	Centre	0.000	0.000	0.000	0.000	0.000	637	[64,114,115]
	South	0.000	0.000	0.000	0.000	0.000	222	[58,59,62,114,115]
Slovakia		0.000	0.000	0.000	0.000	0.000	581	[116,117]
Slovenia		0.000	0.000	0.000	0.000	0.000	233	[66,118]
Sweden		0.296	0.000	0.000	0.000	0.000	338	[44,107], Unpub
Switzerland		0.000	0.000	0.000	0.000	0.000	228	[119,120]
Ukraine		0.000	0.000	0.000	0.000	0.000	110	[39], Unpub
Wales		0.000	0.000	0.000	0.000	0.000	92	[77]

Africa			**2.704**	**0.555**	**0.215**	**0.466**	**0.017**	**5585**	
	Algeria		0.000	0.000	0.000	0.000	0.000	125	[62,115]
	Cameroon		0.000	0.000	0.000	0.000	0.000	649	[121,122], Unpub
	Chad		0.000	0.000	0.000	0.000	0.000	14	[123]
	Egypt		3.129	0.544	0.000	0.000	0.000	735	
		Berbers	2.564	0.000	0.000	0.000	0.000	78	[124]
		non-Berbers	3.196	0.609	0.000	0.000	0.000	657	[125,126], Unpub
	Eritrea		18.349	0.000	7.339	1.835	0.000	109	Unpub
	Ethiopia		9.174	1.988	0.612	3.364	0.000	654	[51,127-129], Unpub
	Guinea		0.000	0.000	0.000	0.000	0.000	11	[130]
	Kenya		1.180	0.337	0.000	0.000	0.000	593	[128,131,132]
	Libya		1.256	0.503	0.000	0.000	0.000	398	[133,134]
	Mauritania & Western Sahara		1.802	0.901	0.000	0.000	0.000	111	[115,135]
	Morocco		1.260	0.097	0.000	0.000	0.097	1032	
		Berbers	0.673	0.000	0.000	0.000	0.000	297	[115,124,135-137]
		non-Berbers	1.497	0.136	0.000	0.000	0.136	735	[59,115,135,137,138], Unpub
	Niger		0.000	0.000	0.000	0.000	0.000	33	[123]
	Nigeria		0.000	0.000	0.000	0.000	0.000	115	[123,139]
	Senegal		0.000	0.000	0.000	0.000	0.000	240	[135,140]
	Somalia		7.692	2.564	0.000	1.709	0.000	117	[123,141]
	South East Africa		0.000	0.000	0.000	0.000	0.000	307	[142]
	Sudan		0.000	0.000	0.000	0.000	0.000	76	[143]
	Tunisia		2.105	0.877	0.000	0.000	0.000	570	
		Berbers	1.935	1.935	0.000	0.000	0.000	155	[144]
		non-Berbers	2.169	0.482	0.000	0.000	0.000	415	[115,138,145]

Unpub = Unpublished data

Table S3. Founder lineages identified when using f1 and f2 criteria from the Fertile Crescent (including Levant, Iran, Iraq), and South Caucasus and Arabian Peninsula to Eastern Africa.

Founder_f1	Haplogroup	n	rho	se	Age estimate	95% c. i. lower b	95% c. i. higher b
**F1**	R0a1b	1	0.00	0.00			
**F2**	R0a1a_58@	3	7.33	2.45	19,968	6,635	34,180
**F3**	R0a1	2	3.50	1.32	9,246	2,343	16,434
**F4**	R0a2b1b1	2	1.00	0.71	2,585	-985	6,244
**F5**	R0a2b1b	1	0.00	0.00			
**F6**	R0a2h1	1	0.00	0.00			
**F7**	R0a2b1	9	4.44	2.01	11,832	1,288	23,021
**F8**	R0a2b2	8	1.13	0.54	2,912	146	5,729
**F9**	R0a2n2	1	0.00	0.00			
**F10**	R0a2g	6	3.50	1.09	9,246	3,522	15,164
**F11**	R0a2a	1	0.00	0.00			
**F12**	R0a2	7	4.57	1.16	12,182	6,006	18,573
**Founder_f2**	**Haplogroup**	**n**	**rho**	**se**	**Age estimate**	**95% c. i. lower b**	**95% c. i. higher b**
**F2**	R0a1a_58@	3	7.33	2.45	19,968	6,635	34,180
**F13**	R0a1_152	1	0.00	0.00			
**F3**	R0a1	2	3.50	1.32	9,246	2,343	16,434
**F4**	R0a2b1b1	2	1.00	0.71	2,585	-985	6,244
**F14**	R0a2h	1	0.00	0.00			
**F15**	R0a2b	18	4.28	1.33	11,373	4,323	18,708
**F16**	R0a2n	1	0.00	0.00			
**F11**	R0a2a	1	0.00	0.00			
**F12**	R0a2	13	4.54	0.93	12,091	7,146	17,173

Table S4. Founder lineages identified when using f1 and f2 criteria from the Fertile Crescent (including Levant, Iran, Iraq) and South Caucasus to Arabian Peninsula and Eastern Africa.

Founder_f1	Haplogroup	n	rho	se	Age estimate	95% c. i. lower b	95% c. i. higher b
**F1**	R0a1a	40	4.00	0.54	10610	7712	13556
**F2**	R0a1	8	5.25	1.41	14067	6490	21955
**F3**	R0a2o1	1	0.00	0.00			
**F4**	R0a2o1_16304	2	0.50	0.50	1287	-1224	3843
**F5**	R0a2o_16304	1	0.00	0.00			
**F6**	R0a2r	2	1.50	0.87	3895	-505	8425
**F7**	R0a2c	4	3.75	1.44	9926	2416	17771
**F8**	R0a2r	68	5.82	0.72	15674	11769	19659
**F9**	R0a2'3	3	2.67	1.25	6995	570	13680
**F10**	R0a_60.1T	1	0.00	0.00			
**Founder_f2**	**Haplogroup**	**n**	**rho**	**se**	**Age estimate**	**95% c. i. lower b**	**95% c. i. higher b**
**F2**	R0a1	88	5.93	0.98	15979	10627	21480
**F6**	R0a2r	2	1.50	0.87	3895	-505	8425
**F8**	R0a2r	76	5.67	0.66	15246	11663	18894
**F10**	R0a_60.1T	4	3.50	1.27	9246	2589	16167

Table S5. Founder lineages identified when using f1 and f2 criteria from the Fertile Crescent (including Levant and Iraq) and South Caucasus to the Arabian Peninsula.

Founder_f1	Haplogroup	n	rho	se	Age estimate	95% c. i. lower b	95% c. i. higher b
**F1**	R0a1a	35	3.66	0.57	9,673	6,678	12,721
**F2**	R0a1a	7	5.00	1.33	13,370	6,250	20,769
**F3**	R0a2o1	1	0.00	0.00			
**F4**	R0a2o1_16304	2	0.50	0.50	1,287	-1,224	3,843
**F5**	R0a1o_16304	1	0.00	0.00			
**F6**	R0a2r	2	1.50	0.87	3,895	-505	8,425
**F7**	R0a2c	4	3.75	1.44	9,926	2,416	17,771
**F8**	R0a2	32	6.59	0.92	17,854	12,800	23,034
**F9**	R0a2'3	3	2.67	1.25	6,995	570	13,680
**F10**	R0a_60.1T	1	0.00	0.00			
**Founder_f2**	**Haplogroup**	**n**	**rho**	**se**	**Age estimate**	**95% c. i. lower b**	**95% c. i. higher b**
**F2**	R0a1a	77	5.60	0.99	15,039	9,691	20,537
**F6**	R0a2r	2	1.50	0.87	3,895	-505	8,425
**F8**	R0a2'3	40	6.15	0.79	16,595	12,284	21,000
**F10**	R0a_60.1T	4	3.50	1.27	9,246	2,589	16,167

Table S6. Founder lineages identified when using f1 and f2 criteria from the Arabian Peninsula to Fertile Crescent (including Levant, Iran and Iraq) and South Caucasus.

Founder_f1	Haplogroup	n	rho	se	Age estimate	95% c. i. lower b	95% c. i. higher b
**F1**	R0a1a8_152	1	0	0			
**F2**	R0a1a3	1	0	0			
**F3**	R0a1a5	1	0	0			
**F4**	R0a1a	1	0	0			
**F5**	R0a2o1	1	0	0			
**F6**	R0a2d_152	1	0	0			
**F7**	R0a2r	2	2.5	1.118	6,549	793	12,514
**F8**	R0a2n	1	0	0			
**F9**	R0a3	1	0	0			
**F10**	R0a2'3	1	0	0			
**F11**	R0a_60.1T	2	5.5	1.658	14,766	5,880	24,077
**F12**	R0	1	0	0			
**Founder_f2**	**Haplogroup**	**n**	**rho**	**se**	**Age estimate**	**95% c. i. lower b**	**95% c. i. higher b**
**F3**	R0a1a5	1	0	0			
**F13**	R0a1a_152	1	0	0			
**F4**	R0a1a	2	4.5	1.5	11,985	4,053	20,275
**F14**	R0a2o1_16304	1	0	0			
**F9**	R0a3	1	0	0			
**F15**	R0a2'3	4	4.25	1.09	11,296	5,520	17,263
**F10**	R0a2'3	1	0	0			
**F11**	R0a_60.1T	2	5.5	1.658	14,766	5,880	24,077

Table S7. Founder lineages identified when using f1 and f2 criteria from the Arabian Peninsula to Fertile Crescent (including Levant and Iraq) and South Caucasus.

Founder_f1	Haplogroup	n	rho	se	Age estimate	95% c. i. lower b	95% c. i. higher b
**F1**	R0a1a	1	0.00	0.00			
**F2**	R0a2o	1	0.00	0.00			
**F3**	R0a2r	2	2.50	1.12	6,549	793	12,514
**F4**	R0a_60.1T	2	5.50	1.66	14,766	5,880	24,077
**F5**	R0a'b	1	0.00	0.00			
**Founder_f2**	**Haplogroup**	**n**	**rho**	**se**	**Age estimate**	**95% c. i. lower b**	**95% c. i. higher b**
**F1**	R0a1a	1	0.00	0.00			
**F6**	R0a2o_16304	1	0.00	0.00			
**F7**	R0a2	2	2.50	1.12	6,549	793	12,514
**F4**	R0a'b	2	5.50	1.66	14,766	5,880	24,077

Table S8. Founder lineages identified when using f1 and f2 criteria from the Fertile Crescent (including Levant, Iran and Iraq), North Africa, the Arabian Peninsula and South Caucasus to India and Pakistan.

Founder_f1	Haplogroup	n	rho	se	Age estimate	95% c. i. lower b	95% c. i. higher b
**F1**	R0a2d_152	2	2.50	1.12	6,549	793	12,514
**F2**	R0a2d	2	4.50	1.80	11,985	2,498	21,989
**F3**	R0a6	10	0.80	0.35	2,065	310	3,840
**Founder_f2**	**Haplogroup**	**n**	**rho**	**se**	**Age estimate**	**95% c. i. lower b**	**95% c. i. higher b**
**F1**	R0a2d_152	2	2.50	1.12	6,549	793	12,514
**F2**	R0a2d	2	4.50	1.80	11,985	2,498	21,989
**F4**	R0a_60.1T	10	11.80	3.33	33,165	14,106	53,645

Table S9. Founder lineages identified when using f1 and f2 criteria from the Fertile Crescent (including Levant, Iran and Iraq), North Africa, the Arabian Peninsula and South Caucasus to Europe.

Founder_f1	Haplogroup	n	rho	se	Age estimate	95% c. i. lower b	95% c. i. higher b
**F1**	R0a1a3a	1	0.00	0.00			
**F2**	R0a1a7	1	0.00	0.00			
**F3**	R0a1a_152	1	0.00	0.00			
**F4**	R0a1a	1	0.00	0.00			
**F5**	R0a2r_14110	1	0.00	0.00			
**F6**	R0a2n2	1	0.00	0.00			
**F7**	R0a2n1	3	0.00	0.00			
**F8**	R0a2a1	1	0.00	0.00			
**F9**	R0a2d	2	0.00	0.00			
**F10**	R0a2f	1	0.00	0.00			
**F11**	R0a2r	9	4.11	1.43	10,915	3,406	18,751
**F12**	R0a2a	2	2.50	1.12	6,549	793	12,514
**F13**	R0a2	4	3.75	1.03	9,926	4,501	15,525
**F14**	R0a5	1	0.00	0.00			
**F15**	R0a_60.1T	5	3.60	1.47	9,518	1,855	17,532
**F16**	R0b1	1	0.00	0.00			
**F17**	R0a	2	0.00	0.00			
**F18**	R0b	1	0.00	0.00			
**F19**		1	0.00	0.00			
**Founder_f2**	**Haplogroup**	**n**	**rho**	**se**	**Age estimate**	**95% c. i. lower b**	**95% c. i. higher b**
**F20**	R0a1a3	1	0.00	0.00			
**F3**	R0a1a_152	1	0.00	0.00			
**F4**	R0a1a	2	1.50	0.87	3,895	-505	8,425
**F10**	R0a2f	1	0.00	0.00			
**F11**	R0a2r	10	4.30	1.31	11,434	4,520	18,621
**F21**	R0a2n	4	1.50	0.61	3,895	770	7,085
**F12**	R0a2a	3	2.33	0.88	6,103	1,558	10,780
**F13**	R0a2	6	5.17	1.17	13,835	7,568	20,313
**F15**	R0a_60.1T	6	4.67	1.33	12,446	5,358	19,815
**F17**	R0a	3	16.00	2.67	46,181	30,069	63,084

Figure S1. Phylogenetic network of HVS-I variation in haplogroup R0a'b. Links are labelled with the nucleotide position for each transition variant, less 16,000, with transversions indicated by the base change and reversions towards the root with suffix @.

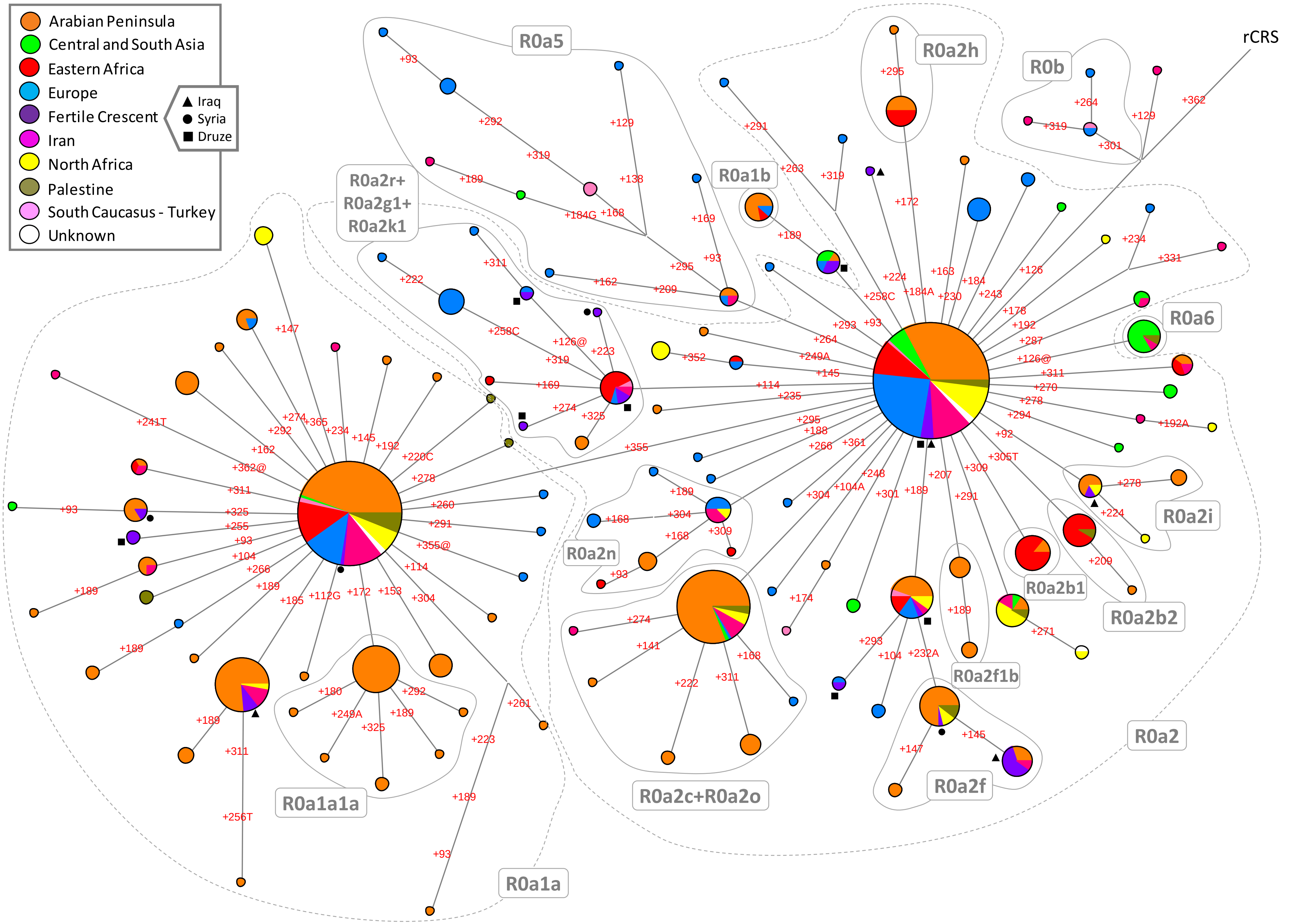

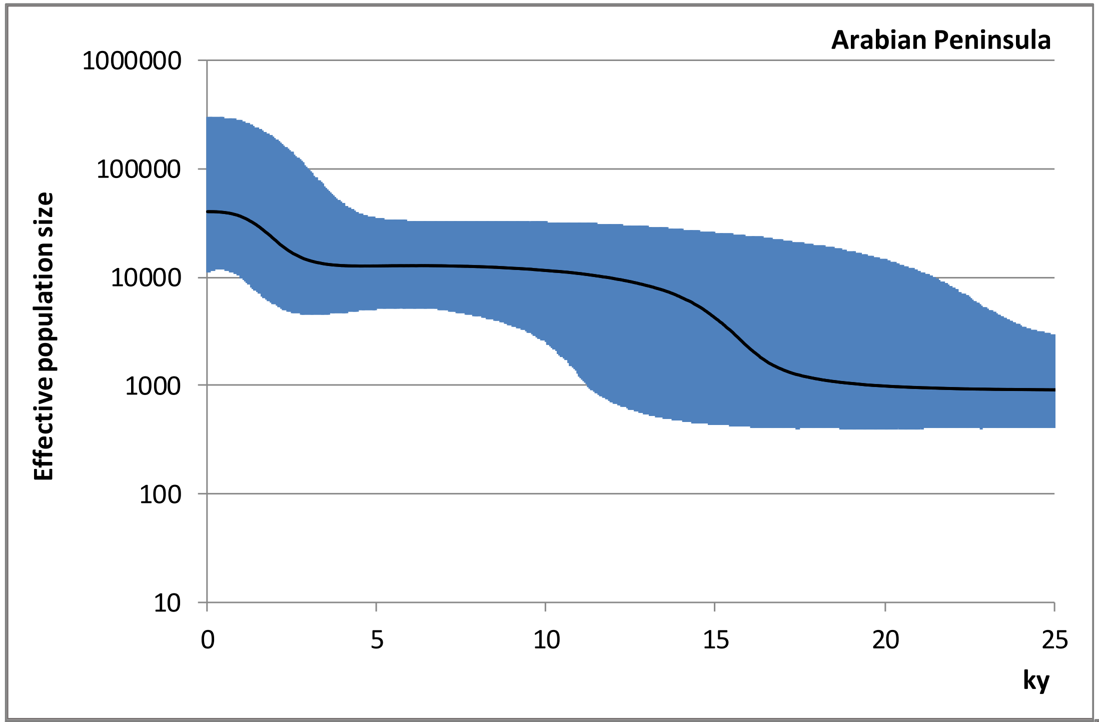

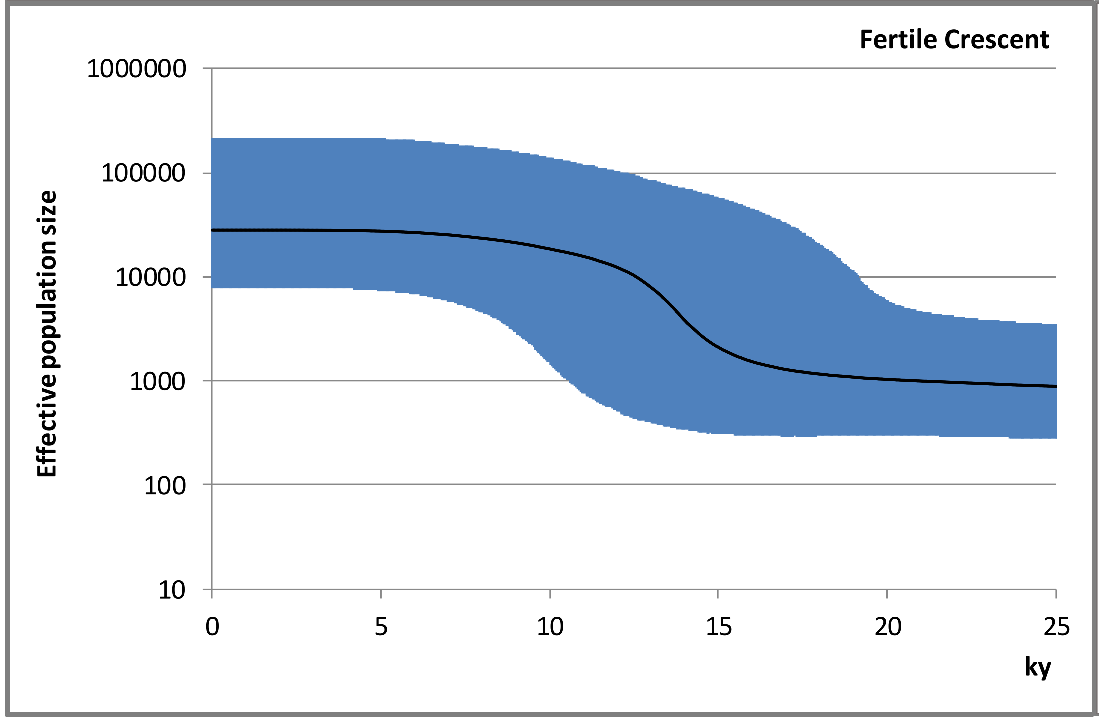

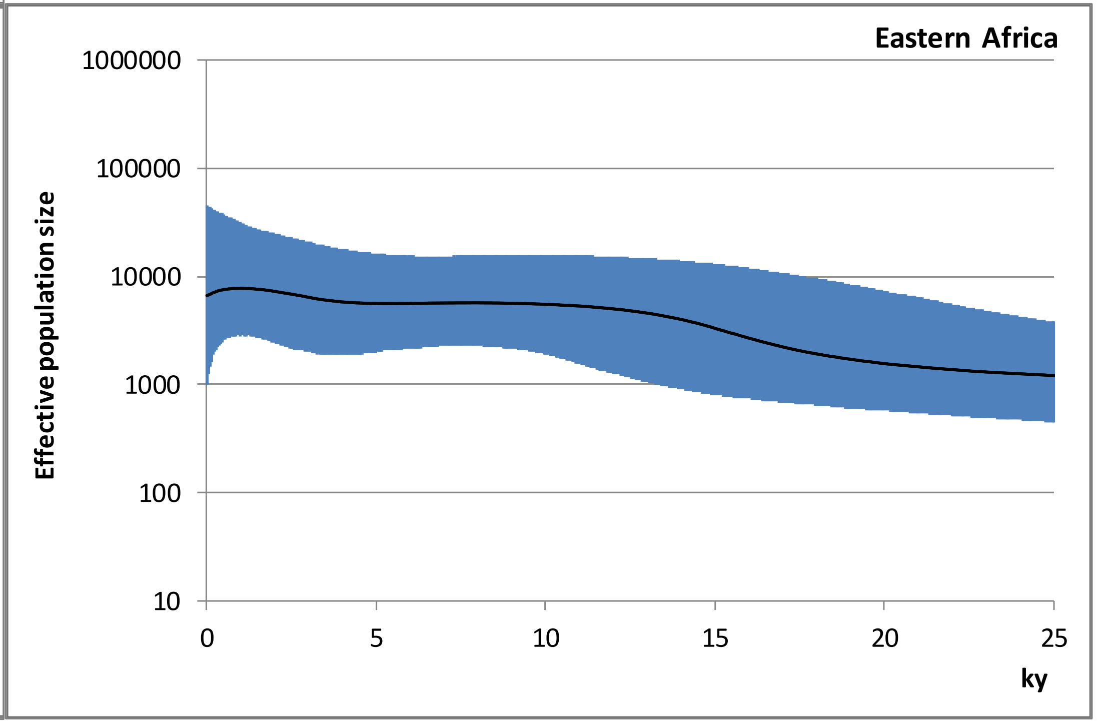

Figure S2. Bayesian skyline plots (BSPs) of R0a samples from the Arabian Peninsula, Fertile Crescent (including the Levant, Iraq and Iran) and Eastern Africa. The thick solid line is the median estimate and the shading shows the 95% highest posterior density limits. The time axis is limited to 25 ka, beyond that time the curves remain linear.

References

1 Lippold, S. *et al.* Human paternal and maternal demographic histories: insights from high-resolution Y chromosome and mtDNA sequences. *Investig Genet* **5,** 13 (2014).

2 Zheng, H.-X., Qin, Z.-D., Jin, L. & Jin, L. *The mitochondrial DNA diversity of HGDP populations* (2014).

3 Cerný, V. *et al.* Internal diversification of mitochondrial haplogroup R0a reveals post-last glacial maximum demographic expansions in South Arabia. *Mol Biol Evol* **28,** 71-78 (2011).

4 Fendt, L. *et al.* Accumulation of mutations over the entire mitochondrial genome of breast cancer cells obtained by tissue microdissection. *Breast Cancer Res Treat* **128,** 327-336 (2011).

5 Derenko, M. *et al.* Complete mitochondrial DNA diversity in Iranians. *PLoS One* **8,** e80673 (2013).

6 Abu-Amero, K. K., Larruga, J. M., Cabrera, V. M. & González, A. M. Mitochondrial DNA structure in the Arabian Peninsula. *BMC Evol Biol* **8,** 45 (2008).

7 Raule, N. *et al.* The co-occurrence of mtDNA mutations on different oxidative phosphorylation subunits, not detected by haplogroup analysis, affects human longevity and is population specific. *Aging cell* **13,** 401-407 (2014).

8 Behar, D. M. *et al.* A "Copernican" reassessment of the human mitochondrial DNA tree from its root. *Am J Hum Genet* **90,** 675-684 (2012).

9 Costa, M. D. *et al.* Data from complete mtDNA sequencing of Tunisian centenarians: testing haplogroup association and the "golden mean" to longevity. *Mech Ageing Dev* **130,** 222-226 (2009).

10 Gasparre, G. *et al.* Disruptive mitochondrial DNA mutations in complex I subunits are markers of oncocytic phenotype in thyroid tumors. *Proc Natl Acad Sci U S A* **104,** 9001-9006 (2007).

11 Behar, D. M. *et al.* Counting the founders: the matrilineal genetic ancestry of the Jewish Diaspora. *PLoS One* **3,** e2062 (2008).

12 Achilli, A. *et al.* Mitochondrial DNA backgrounds might modulate diabetes complications rather than T2DM as a whole. *PLoS One* **6,** e21029 (2011).

13 Greenspan, B. *Family Tree DNA - Genealogy by Genetics, Ltd.* (2007) Available at: https://www.familytreedna.com/. (Acessed: 30th May 2015).

14 Achilli, A. *et al.* The molecular dissection of mtDNA haplogroup H confirms that the Franco-Cantabrian glacial refuge was a major source for the European gene pool. *Am J Hum Genet* **75,** 910-918 (2004).

15 Kovacevic, L. *et al.* Standing at the gateway to Europe--the genetic structure of Western balkan populations based on autosomal and haploid markers. *PLoS One* **9,** e105090 (2014).

16 Abecasis, G. R. *et al.* An integrated map of genetic variation from 1,092 human genomes. *Nature* **491,** 56-65 (2012).

17 Palanichamy, M. G. *et al.* Phylogeny of mitochondrial DNA macrohaplogroup N in India, based on complete sequencing: implications for the peopling of South Asia. *Am J Hum Genet* **75,** 966-978 (2004).

18 Cardoso, S. *et al.* The expanded mtDNA phylogeny of the Franco-Cantabrian region upholds the pre-Neolithic genetic substrate of Basques. *PLoS One* **8,** e67835 (2013).

19 Schönberg, A., Theunert, C., Li, M., Stoneking, M. & Nasidze, I. High-throughput sequencing of complete human mtDNA genomes from the Caucasus and West Asia: high diversity and demographic inferences. *Eur J Hum Genet* **19,** 988-994 (2011).

20 Hartmann, A. *et al.* Validation of microarray-based resequencing of 93 worldwide mitochondrial genomes. *Hum Mutat* **30,** 115-122 (2009).

21 Gomez-Carballa, A. *et al.* Indian signatures in the westernmost edge of the European Romani diaspora: new insight from mitogenomes. *PLoS One* **8,** e75397 (2013).

22 Irwin, J. A. *et al.* The mtDNA composition of Uzbekistan: a microcosm of Central Asian patterns. *Int J Legal Med* **124,** 195-204 (2010).
23 Yao, Y. G., Kong, Q. P., Wang, C. Y., Zhu, C. L. & Zhang, Y. P. Different matrilineal contributions to genetic structure of ethnic groups in the silk road region in china. *Mol Biol Evol* **21,** 2265-2280 (2004).
24 Yao, Y. G., Lü, X. M., Luo, H. R., Li, W. H. & Zhang, Y. P. Gene admixture in the silk road region of China: evidence from mtDNA and melanocortin 1 receptor polymorphism. *Genes Genet Syst* **75,** 173-178 (2000).
25 Yao, Y. G. *et al.* Genetic relationship of Chinese ethnic populations revealed by mtDNA sequence diversity. *Am J Phys Anthropol* **118,** 63-76 (2002).
26 Yao, Y. G., Kong, Q. P., Bandelt, H. J., Kivisild, T. & Zhang, Y. P. Phylogeographic differentiation of mitochondrial DNA in Han Chinese. *Am J Hum Genet* **70,** 635-651 (2002).
27 Cordaux, R. *et al.* Mitochondrial DNA analysis reveals diverse histories of tribal populations from India. *Eur J Hum Genet* **11,** 253-264 (2003).
28 Kivisild, T. *et al.* Deep common ancestry of indian and western-Eurasian mitochondrial DNA lineages. *Curr Biol* **9,** 1331-1334 (1999).
29 Metspalu, M. *et al.* Most of the extant mtDNA boundaries in south and southwest Asia were likely shaped during the initial settlement of Eurasia by anatomically modern humans. *BMC Genet* **5,** 26 (2004).
30 Roychoudhury, S. *et al.* Genomic structures and population histories of linguistically distinct tribal groups of India. *Hum Genet* **109,** 339-350 (2001).
31 Quintana-Murci, L. *et al.* Where west meets east: the complex mtDNA landscape of the southwest and Central Asian corridor. *Am J Hum Genet* **74,** 827-845 (2004).
32 Mountain, J. L. *et al.* Demographic history of India and mtDNA-sequence diversity. *Am J Hum Genet* **56,** 979-992 (1995).
33 Bamshad, M. J. *et al.* Female gene flow stratifies Hindu castes. *Nature* **395,** 651-652 (1998).
34 Rakha, A. *et al.* Forensic and genetic characterization of mtDNA from Pathans of Pakistan. *Int J Legal Med* **125,** 841-848 (2011).
35 Comas, D. *et al.* Trading genes along the silk road: mtDNA sequences and the origin of central Asian populations. *Am J Hum Genet* **63,** 1824-1838 (1998).
36 Kolman, C. J., Sambuughin, N. & Bermingham, E. Mitochondrial DNA analysis of Mongolian populations and implications for the origin of New World founders. *Genetics* **142,** 1321-1334 (1996).
37 Derenko, M. *et al.* Phylogeographic analysis of mitochondrial DNA in northern Asian populations. *Am J Hum Genet* **81,** 1025-1041 (2007).
38 Shields, G. F. *et al.* mtDNA sequences suggest a recent evolutionary divergence for Beringian and northern North American populations. *Am J Hum Genet* **53,** 549-562 (1993).
39 Malyarchuk, B. A. & Derenko, M. V. Mitochondrial DNA variability in Russians and Ukrainians: implication to the origin of the Eastern Slavs. *Ann Hum Genet* **65,** 63-78 (2001).
40 Schurr, T. G., Sukernik, R. I., Starikovskaya, Y. B. & Wallace, D. C. Mitochondrial DNA variation in Koryaks and Itel'men: population replacement in the Okhotsk Sea-Bering Sea region during the Neolithic. *Am J Phys Anthropol* **108,** 1-39 (1999).
41 Starikovskaya, Y. B., Sukernik, R. I., Schurr, T. G., Kogelnik, A. M. & Wallace, D. C. mtDNA diversity in Chukchi and Siberian Eskimos: implications for the genetic history of Ancient Beringia and the peopling of the New World. *Am J Hum Genet* **63,** 1473-1491 (1998).
42 Macaulay, V. *et al.* The emerging tree of West Eurasian mtDNAs: a synthesis of control-region sequences and RFLPs. *Am J Hum Genet* **64,** 232-249 (1999).
43 Shlush, L. I. *et al.* The Druze: a population genetic refugium of the Near East. *PLoS One* **3,** e2105 (2008).
44 Richards, M. *et al.* Tracing European founder lineages in the Near Eastern mtDNA pool. *Am J Hum Genet* **67,** 1251-1276 (2000).

45 González, A. M. *et al.* Mitochondrial DNA variation in Jordanians and their genetic relationship to other Middle East populations. *Ann Hum Biol* **35,** 212-231 (2008).
46 Scheible, M. *et al.* Mitochondrial DNA control region variation in a Kuwaiti population sample. *Forensic Sci Int Genet* **5,** e112-113 (2011).
47 Comas, D., Calafell, F., Bendukidze, N., Fañanás, L. & Bertranpetit, J. Georgian and kurd mtDNA sequence analysis shows a lack of correlation between languages and female genetic lineages. *Am J Phys Anthropol* **112,** 5-16 (2000).
48 Alshamali, F., Brandstätter, A., Zimmermann, B. & Parson, W. Mitochondrial DNA control region variation in Dubai, United Arab Emirates. *Forensic Sci Int Genet* **2,** e9-10 (2008).
49 Di Rienzo, A. & Wilson, A. C. Branching pattern in the evolutionary tree for human mitochondrial DNA. *Proc Natl Acad Sci U S A* **88,** 1597-1601 (1991).
50 Cerný, V. *et al.* Regional differences in the distribution of the sub-Saharan, West Eurasian, and South Asian mtDNA lineages in Yemen. *Am J Phys Anthropol* **136,** 128-137 (2008).
51 Non, A. L., Al-Meeri, A., Raaum, R. L., Sanchez, L. F. & Mulligan, C. J. Mitochondrial DNA reveals distinct evolutionary histories for Jewish populations in Yemen and Ethiopia. *Am J Phys Anthropol* **144,** 1-10 (2011).
52 Cerný, V. *et al.* Out of Arabia-the settlement of island Soqotra as revealed by mitochondrial and Y chromosome genetic diversity. *Am J Phys Anthropol* **138,** 439-447 (2009).
53 Vernesi, C. *et al.* Genetic characterization of the body attributed to the evangelist Luke. *Proc Natl Acad Sci U S A* **98,** 13460-13463 (2001).
54 Di Benedetto, G. *et al.* DNA diversity and population admixture in Anatolia. *Am J Phys Anthropol* **115,** 144-156 (2001).
55 Alfonso-Sánchez, M. A. *et al.* Sequence polymorphisms of the mtDNA control region in a human isolate: the Georgians from Swanetia. *J Hum Genet* **51,** 429-439 (2006).
56 Brandstätter, A., Niederstätter, H., Pavlic, M., Grubwieser, P. & Parson, W. Generating population data for the EMPOP database - an overview of the mtDNA sequencing and data evaluation processes considering 273 Austrian control region sequences as example. *Forensic Sci Int* **166,** 164-175 (2007).
57 Parson, W., Parsons, T. J., Scheithauer, R. & Holland, M. M. Population data for 101 Austrian Caucasian mitochondrial DNA d-loop sequences: application of mtDNA sequence analysis to a forensic case. *Int J Legal Med* **111,** 124-132 (1998).
58 Picornell, A., Gómez-Barbeito, L., Tomàs, C., Castro, J. A. & Ramon, M. M. Mitochondrial DNA HVRI variation in Balearic populations. *Am J Phys Anthropol* **128,** 119-130 (2005).
59 Falchi, A. *et al.* Genetic history of some western Mediterranean human isolates through mtDNA HVR1 polymorphisms. *J Hum Genet* **51,** 9-14 (2006).
60 Alfonso-Sánchez, M. A. *et al.* Mitochondrial DNA haplogroup diversity in Basques: a reassessment based on HVI and HVII polymorphisms. *Am J Hum Biol* **20,** 154-164 (2008).
61 Bertranpetit, J. *et al.* Human mitochondrial DNA variation and the origin of Basques. *Ann Hum Genet* **59,** 63-81 (1995).
62 Côrte-Real, H. B. *et al.* Genetic diversity in the Iberian Peninsula determined from mitochondrial sequence analysis. *Ann Hum Genet* **60,** 331-350 (1996).
63 Richard, C. *et al.* An mtDNA perspective of French genetic variation. *Ann Hum Biol* **34,** 68-79 (2007).
64 Prieto, L. *et al.* The GHEP-EMPOP collaboration on mtDNA population data--A new resource for forensic casework. *Forensic Sci Int Genet* **5,** 146-151 (2011).
65 Decorte, R., Jehaes, E., Xiao, F. X. & Cassiman, J. J. in *Advances in Forensic Haemogenetics 6* 497-503 (Springer-Verlag, 1996).
66 Malyarchuk, B. A. *et al.* Mitochondrial DNA variability in Bosnians and Slovenians. *Ann Hum Genet* **67,** 412-425 (2003).
67 Babalini, C. *et al.* The population history of the Croatian linguistic minority of Molise (southern Italy): a maternal view. *Eur J Hum Genet* **13,** 902-912 (2005).

68 Mikkelsen, M., Sørensen, E., Rasmussen, E. M. & Morling, N. Mitochondrial DNA HV1 and HV2 variation in Danes. *Forensic Sci Int Genet* **4,** e87-88 (2010).
69 Helgason, A. *et al.* mtDna and the islands of the North Atlantic: estimating the proportions of Norse and Gaelic ancestry. *Am J Hum Genet* **68,** 723-737 (2001).
70 Lahermo, P. *et al.* The genetic relationship between the Finns and the Finnish Saami (Lapps): analysis of nuclear DNA and mtDNA. *Am J Hum Genet* **58,** 1309-1322 (1996).
71 Hedman, M. *et al.* Finnish mitochondrial DNA HVS-I and HVS-II population data. *Forensic Sci Int* **172,** 171-178 (2007).
72 Dubut, V. *et al.* mtDNA polymorphisms in five French groups: importance of regional sampling. *Eur J Hum Genet* **12,** 293-300 (2004).
73 Rousselet, F. & Mangin, P. Mitochondrial DNA polymorphisms: a study of 50 French Caucasian individuals and application to forensic casework. *Int J Legal Med* **111,** 292-298 (1998).
74 Baasner, A., Schäfer, C., Junge, A. & Madea, B. Polymorphic sites in human mitochondrial DNA control region sequences: population data and maternal inheritance. *Forensic Sci Int* **98,** 169-178 (1998).
75 Hofmann, S. *et al.* Population genetics and disease susceptibility: characterization of central European haplogroups by mtDNA gene mutations, correlation with D loop variants and association with disease. *Hum Mol Genet* **6,** 1835-1846 (1997).
76 Pfeiffer, H. *et al.* Expanding the forensic German mitochondrial DNA control region database: genetic diversity as a function of sample size and microgeography. *Int J Legal Med* **112,** 291-298 (1999).
77 Richards, M. *et al.* Paleolithic and neolithic lineages in the European mitochondrial gene pool. *Am J Hum Genet* **59,** 185-203 (1996).
78 Tetzlaff, S., Brandstätter, A., Wegener, R., Parson, W. & Weirich, V. Mitochondrial DNA population data of HVS-I and HVS-II sequences from a northeast German sample. *Forensic Sci Int* **172,** 218-224 (2007).
79 Poetsch, M., Wittig, H., Krause, D. & Lignitz, E. Mitochondrial diversity of a northeast German population sample. *Forensic Sci Int* **137,** 125-132 (2003).
80 Brandstätter, A., Klein, R., Duftner, N., Wiegand, P. & Parson, W. Application of a quasi-median network analysis for the visualization of character conflicts to a population sample of mitochondrial DNA control region sequences from southern Germany (Ulm). *Int J Legal Med* **120,** 310-314 (2006).
81 Lutz, S., Weisser, H. J., Heizmann, J. & Pollak, S. Location and frequency of polymorphic positions in the mtDNA control region of individuals from Germany. *Int J Legal Med* **111,** 67-77 (1998).
82 Irwin, J. *et al.* Mitochondrial control region sequences from northern Greece and Greek Cypriots. *Int J Legal Med* **122,** 87-89 (2008).
83 Brandstätter, A. *et al.* Migration rates and genetic structure of two Hungarian ethnic groups in Transylvania, Romania. *Ann Hum Genet* **71,** 791-803 (2007).
84 Brandstätter, A. *et al.* Mitochondrial DNA control region variation in Ashkenazi Jews from Hungary. *Forensic Sci Int Genet* **2,** e4-6 (2008).
85 Irwin, J. A. *et al.* Development and expansion of high-quality control region databases to improve forensic mtDNA evidence interpretation. *Forensic Sci Int Genet* **1,** 154-157 (2007).
86 Helgason, A. *et al.* Estimating Scandinavian and Gaelic ancestry in the male settlers of Iceland. *Am J Hum Genet* **67,** 697-717 (2000).
87 McEvoy, B., Richards, M., Forster, P. & Bradley, D. G. The Longue Durée of genetic ancestry: multiple genetic marker systems and Celtic origins on the Atlantic facade of Europe. *Am J Hum Genet* **75,** 693-702 (2004).
88 Turchi, C. *et al.* Italian mitochondrial DNA database: results of a collaborative exercise and proficiency testing. *Int J Legal Med* **122,** 199-204 (2008).

89 Vernesi, C., Fuselli, S., Castrì, L., Bertorelle, G. & Barbujani, G. Mitochondrial diversity in linguistic isolates of the Alps: a reappraisal. *Hum Biol* **74,** 725-730 (2002).
90 Achilli, A. *et al.* Mitochondrial DNA variation of modern Tuscans supports the near eastern origin of Etruscans. *Am J Hum Genet* **80,** 759-768 (2007).
91 Francalacci, P., Bertranpetit, J., Calafell, F. & Underhill, P. A. Sequence diversity of the control region of mitochondrial DNA in Tuscany and its implications for the peopling of Europe. *Am J Phys Anthropol* **100,** 443-460 (1996).
92 Vona, G. *et al.* Mitochondrial DNA sequence analysis in Sicily. *Am J Hum Biol* **13,** 576-589 (2001).
93 Pliss, L. *et al.* Mitochondrial DNA portrait of Latvians: towards the understanding of the genetic structure of Baltic-speaking populations. *Ann Hum Genet* **70,** 439-458 (2006).
94 Kasperaviciūte, D., Kucinskas, V. & Stoneking, M. Y chromosome and mitochondrial DNA variation in Lithuanians. *Ann Hum Genet* **68,** 438-452 (2004).
95 Zimmermann, B. *et al.* Mitochondrial DNA control region population data from Macedonia. *Forensic Sci Int Genet* **1,** e4-9 (2007).
96 Grzybowski, T. *et al.* Complex interactions of the Eastern and Western Slavic populations with other European groups as revealed by mitochondrial DNA analysis. *Forensic Sci Int Genet* **1,** 141-147 (2007).
97 Malyarchuk, B. A., Grzybowski, T., Derenko, M. V., Czarny, J. & Miścicka-Sliwka, D. Mitochondrial DNA diversity in the Polish Roma. *Ann Hum Genet* **70,** 195-206 (2006).
98 González, A. M. *et al.* Mitochondrial DNA affinities at the Atlantic fringe of Europe. *Am J Phys Anthropol* **120,** 391-404 (2003).
99 Pereira, L., Prata, M. J. & Amorim, A. Diversity of mtDNA lineages in Portugal: not a genetic edge of European variation. *Ann Hum Genet* **64,** 491-506 (2000).
100 Pereira, L., Cunha, C. & Amorim, A. Predicting sampling saturation of mtDNA haplotypes: an application to an enlarged Portuguese database. *Int J Legal Med* **118,** 132-136 (2004).
101 Malyarchuk, B. A. *et al.* Mitochondrial DNA variability in Poles and Russians. *Ann Hum Genet* **66,** 261-283 (2002).
102 Malyarchuk, B., Derenko, M., Denisova, G. & Kravtsova, O. Mitogenomic diversity in Tatars from the Volga-Ural region of Russia. *Mol Biol Evol* **27,** 2220-2226 (2010).
103 Orekhov, V. *et al.* Mitochondrial DNA sequence diversity in Russians. *FEBS Lett* **445,** 197-201 (1999).
104 Passarino, G. *et al.* Different genetic components in the Norwegian population revealed by the analysis of mtDNA and Y chromosome polymorphisms. *Eur J Hum Genet* **10,** 521-529 (2002).
105 Delghandi, M., Utsi, E. & Krauss, S. Saami mitochondrial DNA reveals deep maternal lineage clusters. *Hum Hered* **48,** 108-114 (1998).
106 Sajantila, A. *et al.* Genes and languages in Europe: an analysis of mitochondrial lineages. *Genome Res* **5,** 42-52 (1995).
107 Tillmar, A. O., Coble, M. D., Wallerström, T. & Holmlund, G. Homogeneity in mitochondrial DNA control region sequences in Swedish subpopulations. *Int J Legal Med* **124,** 91-98 (2010).
108 Zgonjanin, D. *et al.* Sequence polymorphism of the mitochondrial DNA control region in the population of Vojvodina Province, Serbia. *Leg Med (Tokyo)* **12,** 104-107 (2010).
109 Alvarez-Iglesias, V. *et al.* New population and phylogenetic features of the internal variation within mitochondrial DNA macro-haplogroup R0. *PLoS One* **4,** e5112 (2009).
110 Crespillo, M. *et al.* Mitochondrial DNA sequences for 118 individuals from northeastern Spain. *Int J Legal Med* **114,** 130-132 (2000).
111 Salas, A., Comas, D., Lareu, M. V., Bertranpetit, J. & Carracedo, A. mtDNA analysis of the Galician population: a genetic edge of European variation. *Eur J Hum Genet* **6,** 365-375 (1998).

112 Cardoso, S. *et al.* Variability of the entire mitochondrial DNA control region in a human isolate from the Pas Valley (northern Spain). *J Forensic Sci* **55,** 1196-1201 (2010).
113 Maca-Meyer, N. *et al.* Y chromosome and mitochondrial DNA characterization of Pasiegos, a human isolate from Cantabria (Spain). *Ann Hum Genet* **67,** 329-339 (2003).
114 Larruga, J. M., Díez, F., Pinto, F. M., Flores, C. & González, A. M. Mitochondrial DNA characterisation of European isolates: the Maragatos from Spain. *Eur J Hum Genet* **9,** 708-716 (2001).
115 Plaza, S. *et al.* Joining the pillars of Hercules: mtDNA sequences show multidirectional gene flow in the western Mediterranean. *Ann Hum Genet* **67,** 312-328 (2003).
116 Malyarchuk, B. A. *et al.* Mitochondrial DNA variability in Slovaks, with application to the Roma origin. *Ann Hum Genet* **72,** 228-240 (2008).
117 Lehocký, I., Baldovic, M., Kádasi, L. & Metspalu, E. A database of mitochondrial DNA hypervariable regions I and II sequences of individuals from Slovakia. *Forensic Sci Int Genet* **2,** e53-59 (2008).
118 Zupanic Pajnic, I., Balazic, J. & Komel, R. Sequence polymorphism of the mitochondrial DNA control region in the Slovenian population. *Int J Legal Med* **118,** 1-4 (2004).
119 Pult, I. *et al.* Mitochondrial DNA sequences from Switzerland reveal striking homogeneity of European populations. *Biol Chem Hoppe Seyler* **375,** 837-840 (1994).
120 Dimo-Simonin, N., Grange, F., Taroni, F., Brandt-Casadevall, C. & Mangin, P. Forensic evaluation of mtDNA in a population from south west Switzerland. *Int J Legal Med* **113,** 89-97 (2000).
121 Cerny, V., Hajek, M., Cmejla, R., Bruzek, J. & Brdicka, R. mtDNA sequences of Chadic-speaking populations from northern Cameroon suggest their affinities with eastern Africa. *Ann Hum Biol* **31,** 554-569 (2004).
122 Coia, V. *et al.* Brief communication: mtDNA variation in North Cameroon: lack of Asian lineages and implications for back migration from Asia to sub-Saharan Africa. *Am J Phys Anthropol* **128,** 678-681 (2005).
123 Watson, E., Forster, P., Richards, M. & Bandelt, H. J. Mitochondrial footprints of human expansions in Africa. *Am J Hum Genet* **61,** 691-704 (1997).
124 Coudray, C. *et al.* The complex and diversified mitochondrial gene pool of Berber populations. *Ann Hum Genet* **73,** 196-214 (2009).
125 Stevanovitch, A. *et al.* Mitochondrial DNA sequence diversity in a sedentary population from Egypt. *Ann Hum Genet* **68,** 23-39 (2004).
126 Saunier, J. L. *et al.* Mitochondrial control region sequences from an Egyptian population sample. *Forensic Sci Int Genet* **3,** e97-103 (2009).
127 Thomas, M. G. *et al.* Founding mothers of Jewish communities: geographically separated Jewish groups were independently founded by very few female ancestors. *Am J Hum Genet* **70,** 1411-1420 (2002).
128 Boattini, A. *et al.* mtDNA variation in East Africa unravels the history of Afro-Asiatic groups. *Am J Phys Anthropol* **150,** 375-385 (2013).
129 Kivisild, T. *et al.* Ethiopian mitochondrial DNA heritage: tracking gene flow across and around the gate of tears. *Am J Hum Genet* **75,** 752-770 (2004).
130 Pinto, F., Gonzalez, A. M., Hernandez, M., Larruga, J. M. & Cabrera, V. M. Genetic relationship between the Canary Islanders and their African and Spanish ancestors inferred from mitochondrial DNA sequences. *Ann Hum Genet* **60,** 321-330 (1996).
131 Brandstätter, A. *et al.* Mitochondrial DNA control region sequences from Nairobi (Kenya): inferring phylogenetic parameters for the establishment of a forensic database. *Int J Legal Med* **118,** 294-306 (2004).
132 Poloni, E. S. *et al.* Genetic evidence for complexity in ethnic differentiation and history in East Africa. *Ann Hum Genet* **73,** 582-600 (2009).

133 Fadhlaoui-Zid, K. *et al.* Mitochondrial DNA structure in North Africa reveals a genetic discontinuity in the Nile Valley. *Am J Phys Anthropol* **145,** 107-117 (2011).
134 Ottoni, C. *et al.* First genetic insight into Libyan Tuaregs: a maternal perspective. *Ann Hum Genet* **73,** 438-448 (2009).
135 Rando, J. C. *et al.* Mitochondrial DNA analysis of northwest African populations reveals genetic exchanges with European, near-eastern, and sub-Saharan populations. *Ann Hum Genet* **62,** 531-550 (1998).
136 Brakez, Z. *et al.* Human mitochondrial DNA sequence variation in the Moroccan population of the Souss area. *Ann Hum Biol* **28,** 295-307 (2001).
137 Aboukhalid, R. *et al.* Mitochondrial DNA control region variation from samples of the Moroccan population. *Int J Legal Med* **127,** 757-759 (2013).
138 Turchi, C. *et al.* Polymorphisms of mtDNA control region in Tunisian and Moroccan populations: an enrichment of forensic mtDNA databases with Northern Africa data. *Forensic Sci Int Genet* **3,** 166-172 (2009).
139 Vigilant, L., Stoneking, M., Harpending, H., Hawkes, K. & Wilson, A. C. African populations and the evolution of human mitochondrial DNA. *Science* **253,** 1503-1507 (1991).
140 Graven, L. *et al.* Evolutionary correlation between control region sequence and restriction polymorphisms in the mitochondrial genome of a large Senegalese Mandenka sample. *Mol Biol Evol* **12,** 334-345 (1995).
141 Mikkelsen, M. *et al.* Forensic and phylogeographic characterisation of mtDNA lineages from Somalia. *Int J Legal Med* **126,** 573-579 (2012).
142 Salas, A. *et al.* The making of the African mtDNA landscape. *Am J Hum Genet* **71,** 1082-1111 (2002).
143 Krings, M. *et al.* mtDNA analysis of Nile River Valley populations: A genetic corridor or a barrier to migration? *Am J Hum Genet* **64,** 1166-1176 (1999).
144 Fadhlaoui-Zid, K. *et al.* Mitochondrial DNA heterogeneity in Tunisian Berbers. *Ann Hum Genet* **68,** 222-233 (2004).
145 Cherni, L. *et al.* Post-last glacial maximum expansion from Iberia to North Africa revealed by fine characterization of mtDNA H haplogroup in Tunisia. *Am J Phys Anthropol* **139,** 253-260 (2009).
